# Supplementary material for: A 23,000-year-old southern Iberian individual links human groups that lived in Western Europe before and after the Last Glacial Maximum
Source: Nat Ecol Evol. 2023 Mar 1;7(4):597–609. doi: 10.1038/s41559-023-01987-0 (PMC10089921; doi:10.1038/s41559-023-01987-0)
Supplement: Supplementary file 1 — Supplementary Discussion and Figs. 1–11. [file 41559_2023_1987_MOESM1_ESM.pdf]

nature ecology & evolution

Article https://doi.org/10.1038/s41559-023-01987-0

A 23,000-year-old southern Iberian individual links human groups that lived in Western Europe before and after the Last Glacial Maximum

In the format provided by the authors and unedited

Supplementary materials

1. Site descriptions

Cueva del Malalmuerzo

Cueva del Malalmuerzo is a cave site located in Moclín (Granada). The cave system was discovered by Francisco Contreras and Francisco Carrión[1]. The first archaeological surveys, which were performed in 1983, revealed archaeological material ascribed to the Neolithic. During the topography of the cave, many rock art paintings attributed to the Solutrean style were found[2]. In 2016, a small excavation (1m x 0.6m) took place in one of the old archaeological profiles from previous excavations. Two lithostratigraphic units were differentiated. From the lower one, three stratigraphic units were defined and directly dated (VI, V and III from bottom to the top). The radiocarbon dates obtained from VI, V and III units fall into the Magdalenian period and revealed a potential inverted stratigraphy (the older date came from the top unit III). The lithic artifacts recovered from those units were ascribed to the Magdalenian period[3]. From the lower lithostratigraphic units, two human teeth were recovered and analyzed in this study. Both teeth directly dated to the Solutrean period which supports the inverted stratigraphy and their correlation with the rock art figures.

- ***MLZ003*** *(Arch. ID: MALM16 SUP2.1/Individual ID: 4.3.D-3): First lower left premolar (34, following FDI nomenclature), directly dated to 23016-22625 cal BP (18950±50 BP; MAMS-48670)*
- ***MLZ005*** *(Arch. ID: MALM16 Sector A 8.2/Individual ID: 2.1.D-1): Lower left canine (33, following FDI nomenclature) sample directly dated to 22979-22570 cal BP (18880±60 BP; MAMS-48672)*

Cueva de Ardales

Cueva de Ardales is located in the province of Málaga, in the Serrezuela, a mountainous area located between the towns of Ardales and Carratraca, at 565 m.a.s.l. and about 50 km north of the current coastline of the Mediterranean Sea. It is located in Triassic limestone and marble units corresponding to the Bonella-Capellán Unit. Cueva de Ardales is a karstic cave system with 1,597m of interconnected cavities, elongated morphology in an ENE-SW direction, with maximum slopes of 35m and a single entrance currently open to the North of Cerro de la Calinoria, at 599 m.a.s.l. During prehistory the cave had two entrances at least. It is structured in

various chambers, of which the Lower Galleries stand out: Saco Room, Star Room, Calvary Gallery and Labyrinths. Another area of the cave is elevated and known as High Galleries. Inside and near the entrance, there is a heavy/large cone of sediments, which forms part of the stratification and which has been used as a stairway access area since the mid-19th century.

Since its discovery in 1821, the cave system has a long research history with tourist exploitation by Doña Trinidad Grund followed by the integration into prehistoric sciences through the first studies carried out by Henri Breuil and Miguel Such from 1918, who documented the first panels with Paleolithic art[4]. After a long period of abandonment and after the Spanish Civil War, the cave was re-opened in the mid-1980s and various projects were carried out to clean up and document the archaeological record and rock art. It was thus possible to document more than 1,000 painted and engraved motifs, with animals, female human figures, hands, lines, points, and stains. We refer to Cantalejo and colleagues[5,6] for a historiographic synthesis and to numerous studies on the prehistoric art[6–17].

In recent years a General Research Project (GRP) was developed by the authors of this study, with authorization from the Andalusian Government, which allowed the examination and excavation of four areas. Archaeological surveys have obtained a significant chronostratigraphic sequence from the Middle Paleolithic to recent Prehistory (Neolithic and Chalcolithic). Ongoing work by the GRP team includes further archaeological surveys, a variety of chronostratigraphic and interdisciplinary studies on the analysis of archaeological products, and also involves new technologies to study Paleolithic rock art and anthropological remains[18–24].

We sampled individuals from occupation layers in zone 2 at Cueva de Ardales, which were directly dated to the Neolithic and Chalcolithic, and thus surprisingly younger than the stratigraphy had indicated. Ongoing excavations revealed that they were intrusive in a Solutrean layer due to disturbance by an animal burrow[25]. The detailed list of individuals included in this study is reported below:

- ***ADS002*** *(Arch. ID: CA15-Z2, M19b-208.5 064): First lower right incisor (41 following FDI nomenclature) directly dated to 4986-4797 cal BCE/ 6935-6746 cal BP.*
- ***ADS003*** *(Arch. ID: CA16-Z2, M19a-214.36): Third lower left molar (38 following FDI nomenclature). No 1240k data, only MT information.*

- ***ADS004** (Arch. ID: CA16-Z2, M19b-214.2 S64): Third upper right molar (18 following FDI nomenclature) No 1240k data, only MT information.*
- ***ADS005** (Arch. ID: Galerías Bajas Nicho Escalinata): Second lower right molar (47 following FDI nomenclature), directly dated to 5304-5069 cal BCE/ 7253-7018 cal BP (6236±24 BP; MAMS-48675).*
- ***ADS006** (Arch. ID: Galerias Bajas Sala de las Estrellas): First lower left molar (36 following FDI nomenclature). No 1240k data, only MT information.*
- ***ADS007** (Arch. ID: Galerías Altas Ossario principal): Petrous bone. Failed radiocarbon date.*
- ***ADS008** (Arch. ID: Galerías Altas, Sala de la Hornacina): First lower left molar (36 following FDI nomenclature), directly dated to 3341-3093 cal BCE/ 5290-5042 cal BP (4486±21 BP; MAMS-48676).*
- ***ADS009** (Arch. ID: Galerías Altas, Galería de los Huesos): First lower left premolar (44 following FDI nomenclature). No 1240k SNP data, only MT information.*

Necrópolis de las Aguilillas

Las Aguilillas is a necropolis located between the towns of Ardales and Campillos (Málaga), at the confluence of the Guadalhorce, Guadalteba and Turón rivers at 503 m.a.s.l. (maximum level) and distributed in four sectors in the Cerro de Las Aguilillas[26–28]. The necropolis is artificially carved into an Upper Miocene sandstone outcrop, where seven tombs have been documented. These tombs correspond to oval-shaped chambers, which sometimes have niches and entrance corridors, with dimensions between 3.2m and 2.6m and horizontal domes between 1.9m and 1.6m.

One tomb, specifically structure 6, is a megalithic construction formed by an access corridor 7.5m long, a first rectangular chamber and a small corridor through which a second chamber of smaller proportions is accessed. The two chambers have a flat roof made of large horizontal sandstone slabs. The grave goods are very characteristic, with bowls, pots and ceramic vessels of good quality. The lithic industry includes foliaceous points of bifacial carving and flat retouches with a concave base. There are pressure carving blades and sickle elements. An important set of possible peaks for the elaboration of artificial sandstone caves - more than 200 specimens - were documented in a niche. Among the metallic elements there are palmela points and punches[26,27].

This type of necropolis with collective burials is very characteristic of the central zone of Andalusia (the interior of Málaga, the plains of Antequera, and the current countryside of Seville, Córdoba and Jaén) with very defined chronologies between

the end of the 4th and the beginning of the 2nd millennia cal. BCE. However, new direct radiocarbon dates from the individual analyzed from *cave 2* have revealed an older date, suggesting the beginning of funerary use during the Neolithic.
It is interesting to note that in front of the necropolis is the settlement of El Castillón, located about 500m to the east, on the other side of the Guadalteba River, which is considered the town and habitat to which the necropolis is associated[29].

- ***AGS001** (Arch. ID: Necrópolis de Aguilillas/2-C IIA): First upper left incisor (11 following FDI nomenclature) directly dated to 5304-5070 cal BCE/ 7253-7019 cal BP (6237±24 BP; MAMS-48677).*
- ***AGS002** (Arch. ID: Necrópolis de Aguilillas/2-C II -5 220): Third lower left molar (48 following FDI nomenclature). The amount of endogenous DNA was too low to perform any type of capture.*
- ***AGS003** (Arch. ID: Necrópolis de Aguilillas/2-C -5): Second lower left molar (47 following FDI nomenclature). The amount of endogenous DNA was too low to perform any type of capture.*

Necrópolis de los Caserones

El Caserón is a necropolis located in the town of Ardales (Málaga), at the base level of a dam next to the Turón River Turón. The site has single and double burials, made up of a box of slabs, or orthostats, in a quadrangular or rectangular shape with cover slabs, which correspond to the Bronze Age[29,30]. These tombs contain burnished ceramic vessels, with faired shapes, together with metallic elements such as daggers with arsenic copper rivets, swords, quadrangular awls, and silver rings[31]. Culturally, these tombs mark a process of important social hierarchization, with a gradual abandonment of collective burials of Chalcolithic tradition followed by burials that emphasise the individual and smaller families[29,30].
It is interesting to note that these cist necropoles date after the collective burials in the Guadalteba region, such as the one mentioned in Aguilillas. Los Caserones, located next to the Guadalteba and Turón rivers (Las Grajeras, Morenito, Raja del Boquerón, Caserones, Retamar, La Bolina)[30,32], may correspond to the westernmost limit of the El Argar Culture in southern Iberia[33–35]. However, the ^{14}C date obtained from individual CRS002 clearly predates the El Argar Bronze Age horizon.

- ***CRS001** (Arch. ID: Necrópolis de Caserones/CS-83): Second lower left molar (47 following FDI nomenclature). No 1240k SNP data, only MT information.*
- ***CRS002** (Arch. ID: Necrópolis de Caserones/infans II): Second lower right molar (37 following FDI nomenclature) directly dated to 2836-2487 cal BCE/ 4785-4436 cal BP (4058±22 BP; MAMS-48678).*

2. Quality controls

A combination of several quality controls were applied at library and/or individual level to evaluate the authenticity of the aDNA sequences.

2.1 Damage patterns

We used MapDamage (v2.0.6)[36] to explore the damage pattern of each library and the final merged libraries. For example, individual libraries from MLZ showed very similar patterns, with damage at the 5'-end ranging between 0.46 to 0.27. The damage rate at the 5'-end for all libraries is reported in **Supplementary Table 1.4** and shown in **Supplementary Fig. 1a**.

2.2 PMD filtering

We used PMDtools[37] to identify reads with high rates of DNA damage (PDM score > 3) as these are less likely to come from modern DNA contamination. **Supplementary Table 1.5** shows the percentage of retained reads per individual and the number of SNPs covered before and after filtering with PMDtools.

Although there was no indication of contamination using other approaches (see **Supplementary 2.2-2.4**), we nonetheless genotyped PMD- and non-PMD-filtered versions of all merged libraries and replicated the analyses with the two versions of genotype data from the same individual.

2.3 Sex determination

We used sex determination to detect contamination from the opposite sex[38]. Here we calculated the X-ratio and Y-ratio (see methods) in all individual libraries and merged libraries before and after PMD filtering (**Supplementary Fig. 1c, Supplementary Table 1.7**). The scatter plots reflect a clear distinction of genetic (chromosomal) sexes in all individuals (**Supplementary Fig. 1b-1c, Supplementary Table 1.2**). After PMD filtering, the X-ratio and Y-ratio remains similar for all the individuals (**Supplementary Fig. 1c**).

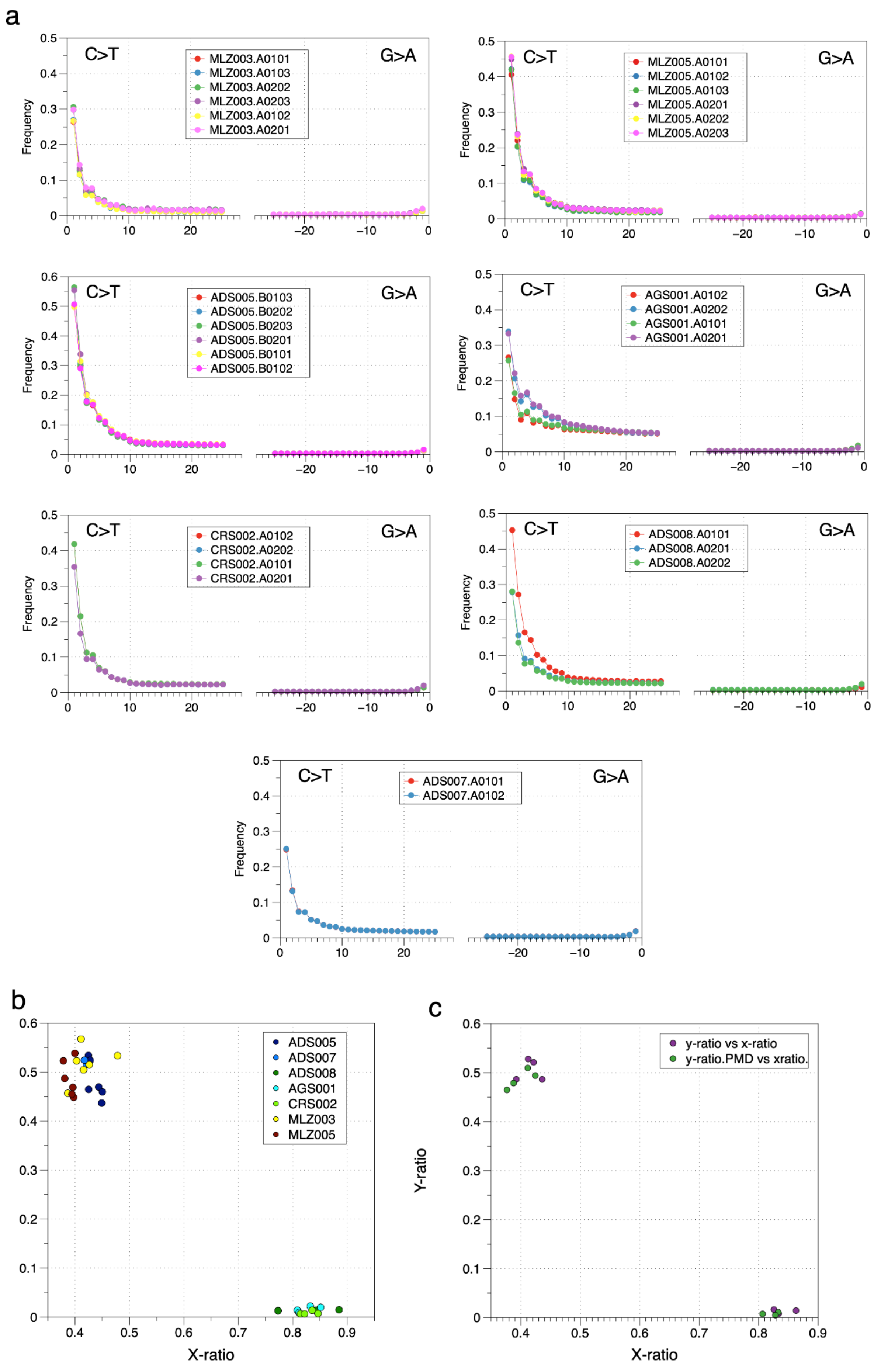
a
C>T
G>A
Frequency
MLZ003.A0101
MLZ003.A0103
MLZ003.A0202
MLZ003.A0203
MLZ003.A0102
MLZ003.A0201
MLZ005.A0101
MLZ005.A0102
MLZ005.A0103
MLZ005.A0201
MLZ005.A0202
MLZ005.A0203
ADS005.B0103
ADS005.B0202
ADS005.B0203
ADS005.B0201
ADS005.B0101
ADS005.B0102
AGS001.A0102
AGS001.A0202
AGS001.A0101
AGS001.A0201
CRS002.A0102
CRS002.A0202
CRS002.A0101
CRS002.A0201
ADS008.A0101
ADS008.A0201
ADS008.A0202
ADS007.A0101
ADS007.A0102
b
ADS005
ADS007
ADS008
AGS001
CRS002
MLZ003
MLZ005
X-ratio
c
y-ratio vs x-ratio
y-ratio.PMD vs xratio.
Y-ratio
X-ratio

Supplementary Fig. 1 I Summary of quality controls. a,Damage rate per single-stranded library and individual at 5' end. **b**, Sex determination using X/autosomes-ratio vs Y/autosomes ratio as indicator of genetic contamination from the opposite sex performed at library level. **c**, Sex determination plot using the X/autosomes-ratio versus Y/autosomes ratio as indicator of genetic contamination from the opposite sex. We show the merged libraries from the same sample and their comparison with the PMD damage-filtered version of each merged library.

2.6 Pairwise mismatch rate (PMR) to identify duplicates and contamination among libraries

Due to the fact that MLZ003 and MLZ005 showed the same uniparental markers (MT and Y-chromosome haplogroups, (**Supplementary Table 1.1-1.2**) and both were different teeth recovered from the same area of excavation, we calculated the PMR[39] to test whether MLZ003 and MLZ005 were in fact the same individual sampled twice. In addition, we also calculated the PMR between libraries to try to identify potential libraries with high levels of contamination, which would result in higher PMR values based on the rationale that background contamination comes from several sporadic sources and thus creates higher levels of PMR.

Following Mittnik et al[40] we calculated a baseline PMR value from the pseudo-haploid 1240k genotyped data using different libraries from the same individual. The median value of the baseline for each sample is reported in **Supplementary Table 1.6** and plotted in **Supplementary Fig. 2a-2b**. From **Supplementary Fig. 2a** we observed that MLZ003 showed high PMR values for some of the pairwise comparisons between libraries which was indicative of background contamination in some of the MLZ003 libraries. When we compared libraries from MLZ003 and MLZ005 the mean PMR value was estimated at 0.15246. This value suggested that MLZ003 and MLZ005 were in fact the same individual, whereas subtle contamination observed in some MLZ003 libraries could have artificially increased the PMR value, as the median PMR value among all pairwise combinations of MLZ003 and MLZ005 was higher than the baseline PMR calculated with different libraries from MLZ005 only (**Fig. S2a**). After removing the problematic MLZ003 libraries, which created a higher PMR (all from extract MLZ003.A01 plus one library from extract MLZ003.A02, see **Supplementary Table 1.6**), the median PMR for MLZ003 and MLZ005 decreased to 0.10387 (**Supplementary Fig. 2b**). A graphical summary of the baselines and PMR values is shown in **Supplementary Fig. 2a-2b**, before and after the exclusion of the contaminated libraries from MLZ003, respectively.

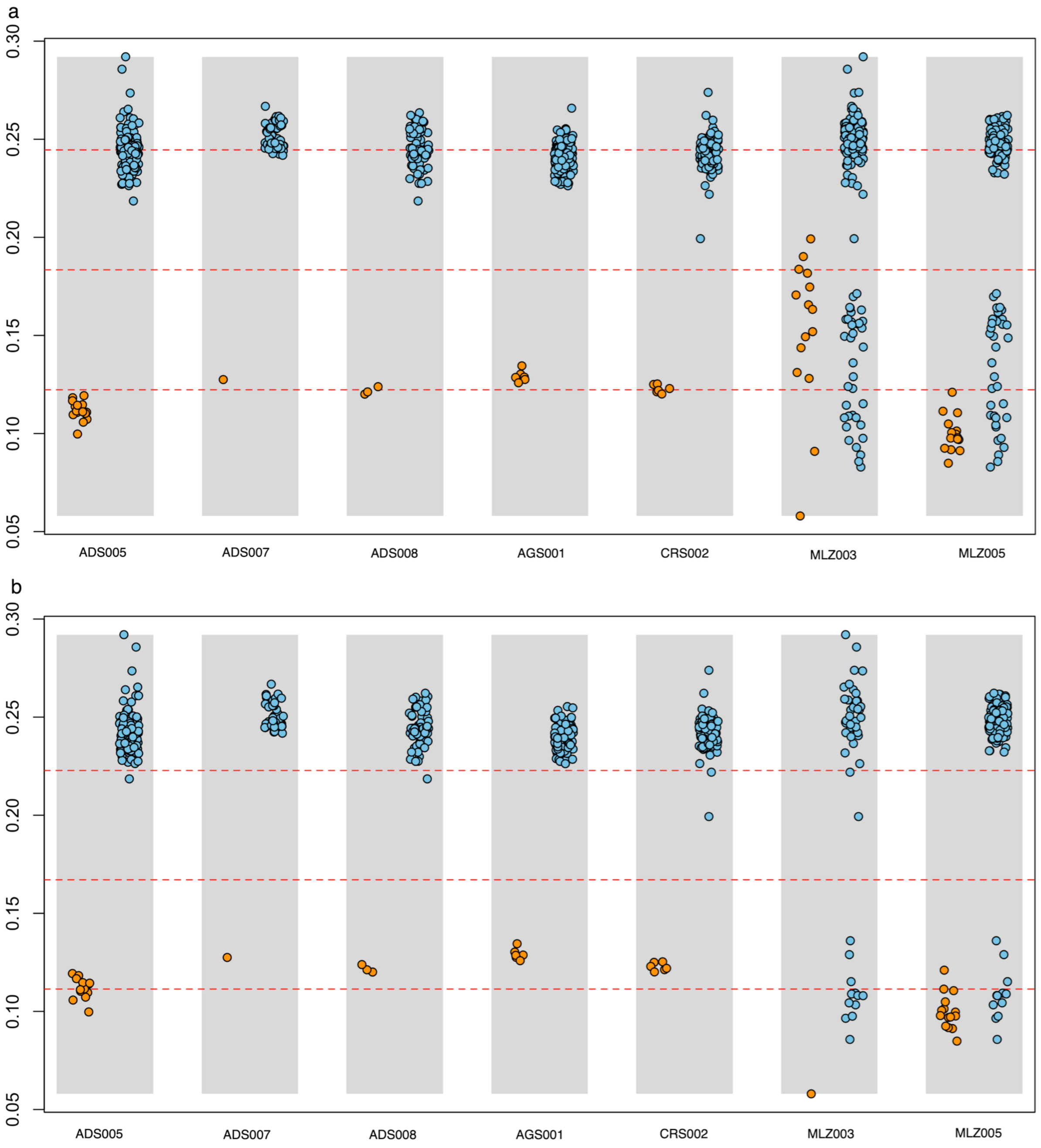

Supplementary Fig. 2 | PMR analysis applied at library level. a, PMR values for each individual calculated from different libraries from the same individual (orange) and the PMR between all libraries from the individuals analyzed in this study (blue). Note that the PMR baseline for MLZ003 showed a large dispersion indicating contamination of some of the libraries (**Table S1.6**). **b,** PMR baseline values after removing the contaminated libraries from MLZ003. Note that MLZ005 values are corresponding with those from MLZ003, indicating that MLZ005 and MLZ003 are the same individual sampled twice (**Table S1.6**).

2.4 X-contamination estimation

We used ANGSD (method 2) to estimate heterozygosity at polymorphic sites on the X chromosome in males[41]. We used the threshold of 100 X-SNPs covered twice to give a confident contamination estimation. As many of our libraries did not reach that threshold, we estimate contamination in the merged bam file which contains all

libraries from the same individual. Following this criteria, only the Paleolithic individual MLZ005 reached 115 X-SNPs covered twice and gave an X-contamination estimation of 0.041 ±0.041. MLZ003 only reached 56 X-SNPs covered twice, respectively, and thus x-contamination estimation was not possible. The nuclear contamination rate of the merged MLZ005003 was estimated at 3.7%. In the rest of the male individuals, X-contamination was measurable and below 0.032 ±0.025 (**Supplementary Table 1.8**).

2.5 Mitochondrial DNA-contamination estimation

We merged several mitogenome-captured libraries (**Supplementary Table 1.9**) to estimate mitochondrial (MT) contamination with ContamMix[42]. For the MLZ003 and MLZ005 merged libraries we reached more than 2000 reads on the MT genome to confidentially estimate contamination levels (**Supplementary Table 1.9**). MLZ003 gave a MT-contamination estimation of 2.5% and MLZ005 of 3.1%, both revealing low levels of MT-contamination. The mitochondrial contamination rate of the merged MLZ005003 was estimated at 5%. For the rest of the individuals, only AGS001 had a rate of contamination of 7.1%, while all others were below 3.9% (**Supplementary Table 1.9**).

2.7 Comparison of standard and PMD-filtered genotype data, and data restricted to transversions (TV).

For the UP individuals, we performed several PCAs to evaluate the deviations in PC-space between standard and PMD-filtered genotype data of MLZ and used these as an indirect quality control, assuming that a highly contaminated individual should plot far from the PMD-filtered version. Additionally, we also restricted the genotyping to transversions only (MLZ005003.TV), and plotted all versions to evaluate whether DNA damage had an effect on the position in PC space.

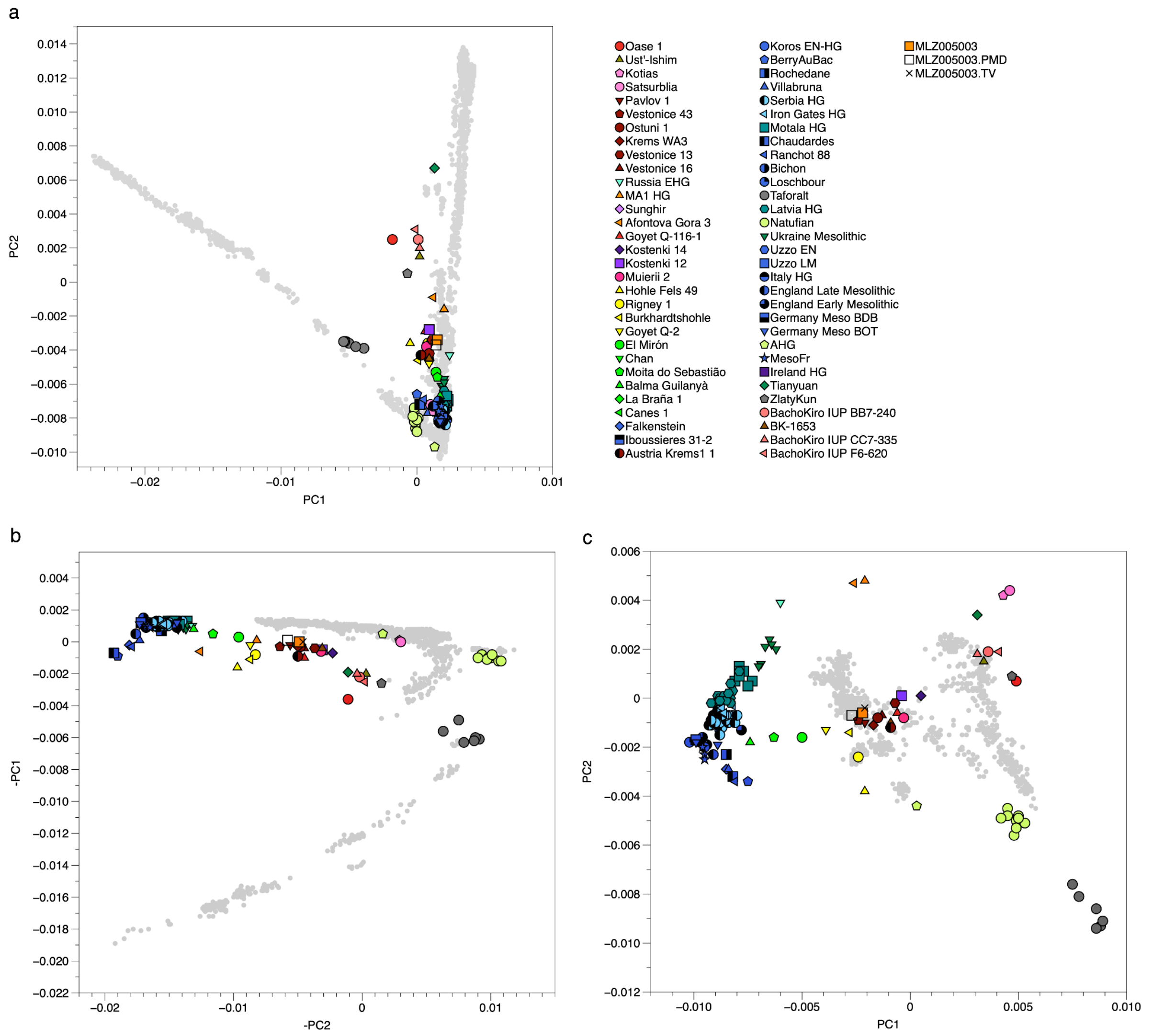

Supplementary Fig. 3 | PCA calculated with modern-day populations (grey points), on which relevant ancient individuals were projected. For MLZ individuals standard, PMD-filtered, and transversions-only genotype data are shown. **a,** Worldwide PCA**; b,** plus PCA with West Eurasian and Sub-Saharan populations ; **c,** West Eurasian PCA.

From the PCA plots we consistently observed that MLZ005003 does not deviate from MLZ005003.PMD or .TV (**Supplementary Fig. 3**). We thus retained individual MLZ005003, but we nonetheless provide separate results for the three versions in all statistics presented in this study.

3. F_3-outgroup statistics

We used f_3*(HG1,HG2; Mbuti)* to explore the genetic affinity among HGs for all pairs of individuals/groups with more than 30,000 SNPs covered in the 1240k panel, including the newly reported MLZ005003. Results are summarized as a heatmap (**Supplementary Fig. 4**), on which we could differentiate the previously defined HG

clusters: Gravettian, Magdalenian (including Iberian HG), WHG, CHG, Mal'ta, and Bacho Kiro IUP. We observed that MLZ005003 falls within the Magdalenian-associated *Goyet Q2* and *Iberian HG* clusters, but outside the genetic variability of the preceding central European Gravettian-associated individuals of the *Věstonice* cluster. We then inverted the f_3-outgroups matrix to a distance matrix calculating 1-f_3*(HG1,HG2; Mbuti)* for all the pairwise comparisons, and applied a MDS analysis shown in **Fig. 2**.

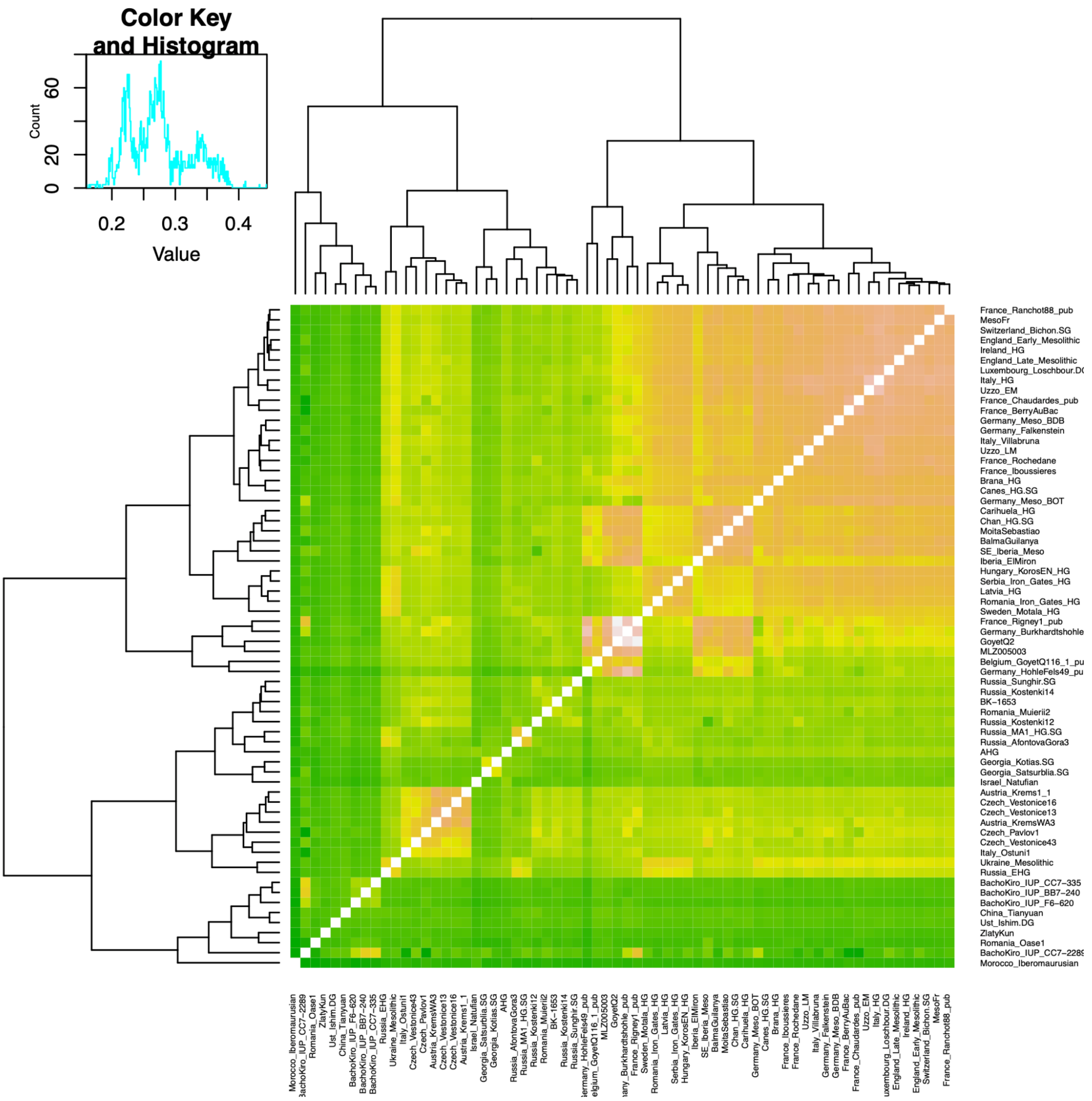

Supplementary Fig. 4 | Heatmap based on f_3-outgroup statistics of the form f_3(HG1, HG2; Mbuti) including HG individuals with more than 30,000 SNPs in all possible pairwise combinations. Warm colors (from yellow to orange) reflect shorter genetic distances (higher f_3-values) and green colors reflect higher genetic distances (lower f_3-values).

4. Relationship of MLZ to other ancient individuals

First, we used f_4-statistics of the form f_4*(MLZ, Kostenki14, test, Mbuti)* to explore the relationship of MLZ to other ancient individuals (**Supplementary Fig. 5a**, **Supplementary Table 2.5**). Here, Kostenki14 was chosen as the baseline for European genetic ancestry as Kostenki14 harbors ancestry that contributed to all later prehistoric and present-day Europeans[43]. Positive f_4-values of this test indicate an excess of shared alleles of MLZ with other ancient populations. We observed that MLZ had high levels of allele sharing with the *Goyet Q2* cluster, Goyet Q116-1, Iberian HGs, WHGs, as well as Natufians and Morocco Iberomaurusian (**Supplementary Fig. 5a, Supplementary Table 2.5**). We did not observe any excess of allele sharing with CHG or central European Gravettian-associated individuals compared to the ancient European ancestry represented by Kostenki14 (**Supplementary Fig. 5a, Supplementary Table 2.5**).

We did not detect excess affinity between MLZ and Gravettian-associated individuals from central Europe with respect to the Villabruna cluster or individual Goyet Q116-1 using f_4-statistics of the form f_4(MLZ, Goyet Q116-1/Villabruna, Mbuti). The results suggest genetic discontinuity between the MLZ and individuals associated with the central European Gravettian (*Věstonice* cluster) (**Supplementary Table 2.4**).

Finally, we noticed an excess of shared genetic affinity of MLZ and IUP individuals such as Bacho Kiro IUP and Tianyuan (**Supplementary Fig. 5a, Supplementary Table 2.5**). However, we also noticed that other IUP individuals, such as Ust'Ishim, showed an attraction to MLZ when compared with other Paleolithic and Mesolithic individuals using f_4-statistics of the form f_4*(test, MLZ; Ust'Ishim, Mbuti)* (**Supplementary Fig. 5b; Supplementary Table 2.8**). Negative results suggest that Ust'Ishim does not really represent an evolutionary dead end as was previously suggested[43], but instead contributed to later populations. This has also been shown by Prüfer and colleagues[44] who had tested Ust'Ishim together with Zlatý kůň using a D-statistic of the form *D(Ust'Ishim, ZlatyKun; test, Mbuti)*, which yielded significantly positive D-statistics when European and Asian HGs were tested, indicating that Ust'Ishim had indeed contributed ancestry to later populations. This observation was confirmed by[45], who reported a non-significant attraction of Ust'Ishim to Bacho Kiro and thus modeled Ust'Ishim as an early independent lineage who had received ancestry from Bacho Kiro. In agreement with this finding, using an f_4-statistic of the form f_4*(test, MLZ; Ust'Ishim, Mbuti),* we also report a generally higher affinity between MLZ and Ust'Ishim than other tested HGs (**Supplementary Fig. 5b; Supplementary Table 2.8**). This could be explained by MLZ sharing more ancestry with Goyet Q116-1 (who also received ancestry from Bacho Kiro IUP) and to a lesser extent from

Bacho Kiro IUP directly. However, it is also possible that the populations tested shared ancestry with other much deeper lineages, who had segregated early after the main African-Eurasian split, such as the inferred Basal Eurasian lineage, which would also return negative results due to the shared drift between *test* populations and the outgroup Mbuti (**Supplementary Fig. 5b**). This could also be the case for some WHG, Scandinavian HGs (SHG) and EHG individuals (e.g. Serbia and Romania_Iron_Gates HG, Motala_HG, Russia_EHG, and Latvia HG groups). The latter had received ancestry from CHG, who are enriched in Basal Eurasian ancestry, but this would not explain the significant negative value obtained in f_4*(Sunghir, MLZ; Ust'Ishim, Mbuti)* (**Supplementary Fig. 5b; Supplementary Table 2.8**).

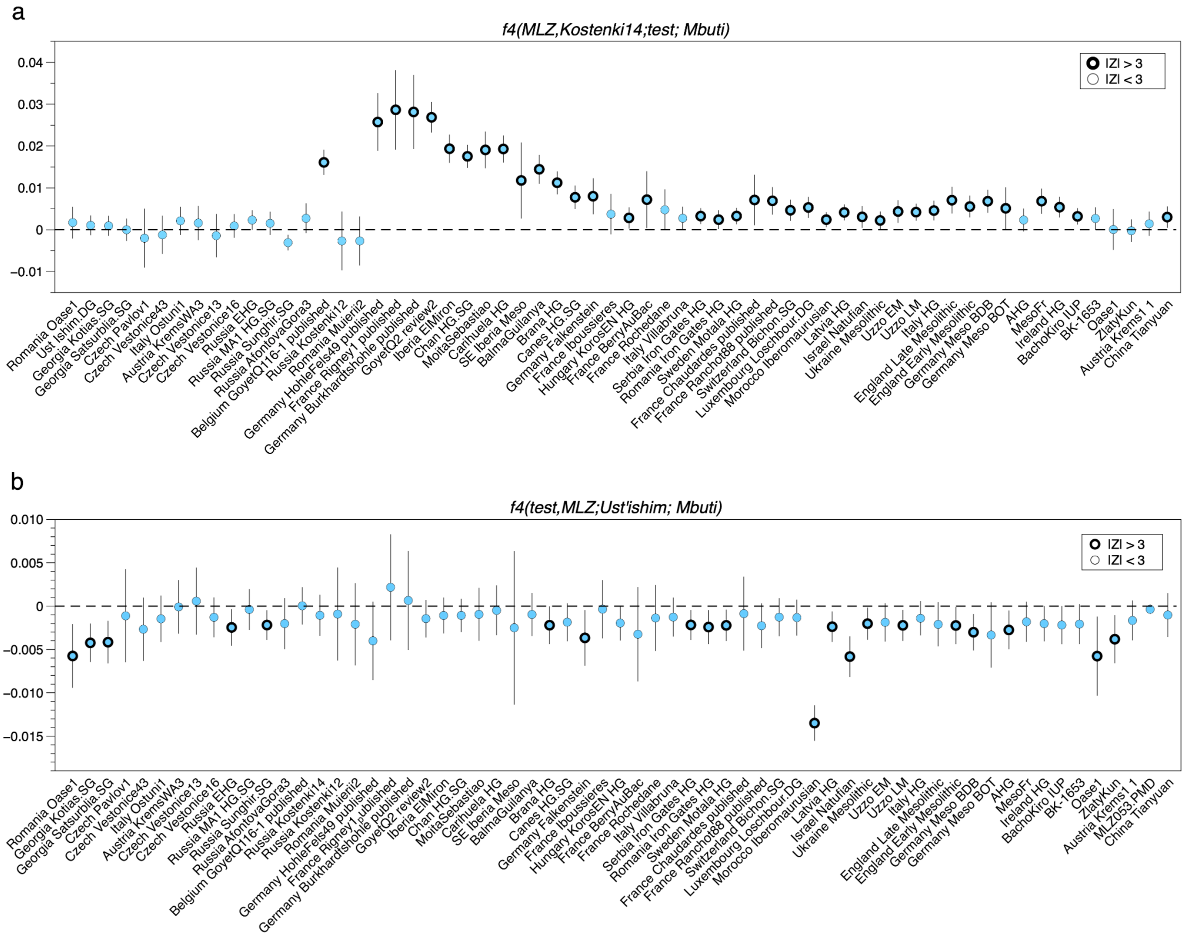

Supplementary Fig. 5 | Genetic affinity of individual MLZ003005. a, Extended version of **Fig. 3** with f_4-statistics of the form f_4*(MLZ, Kostenki14; test, Mbuti)* including HG individuals with more than > 30,000 SNPs (**Table S2.5**). Here, positive f_4-statistics indicate shared drift with MLZ. **b**, f_4-statistics of the form f_4*(test, MLZ; Ust'Ishim, Mbuti)* including HG individuals with more than > 30,000 SNPs (**Supplementary Table S2.8**). Predominantly negative results are indicative of shared ancestry between Ust'Ishim and MZ. This test is significantly negative when the test population received ancestry from a population that split earlier than Ust' Ishim (such as Bacho Kiro, populations with Basal Eurasian ancestry such as CHG, or high levels of Neanderthal ancestry like Oase1) due to their proximity to the African/non-African split represented by Mbuti. For all f_4-statistics, error bars indicate ± 3 SE and were calculated using a weighted block jackknife [46] across all autosomes on the 1240k panel (nsnps = 1150639) and a block size of 5 Mb.; |Z| > 3 points with thicker outline.

5. Traces of Early Asian ancestry

The 40 ka cal BP Tianyuan individual from East Asia was shown to share more ancestry with present-day East Asians and Native Americans than with present-day or ancient Europeans, which led to the conclusion that the split between ancient Asians and Europeans must have happened at least 40 ka ago[47]. Following these findings, the ancestry found in Tianyuan was defined as Early Asian ancestry. Intriguingly, among Paleolithic individuals published by Fu et al.[43], an Aurignacian-associated individual from Goyet cave (in today's Belgium) named Goyet Q116-1 was found to be closely related to ancient Europeans but also shared more alleles with Tianyuan than any other individual in Europe. Yang et al.[48] (now with 1240k SNP capture data) proposed the hypothesis that both Tianyuan and Goyet Q116-1 belonged to a substructured pan-Eurasian population that predated the population split that led to Europeans and Asians. Consequently, both Tianyuan and Goyet Q116-1 shared more ancestry with each other and with an unsampled Eurasian substructured population that did not contribute ancestry to other UP Eurasians. The absence of Denisovan ancestry in Tianyuan (or at least not more than in present-day Asians) made the alternative hypothesis involving a back migration from Asia to Europe during the UP less plausible[48]. Recently, it was shown that individuals associated with the Early Aurignacian from Bacho Kiro in today's Bulgaria also show affinity to Tianyuan and Goyet-Q116-1, and thus represent a population which contributed ancestry to later populations with which Tianyuan and Goyet Q116-1 share their ancestry with[45].

Using an *f4-statistics* of the form f_4*(MLZ, Kostenki14; UP, Mbuti)* (**Fig. 3a, Supplementary Table 2.5**), we can show that the MLZ individual from Southern Iberia shares ancestry with Bacho Kiro IUP, Tianyuan and Goyet Q116-1. These results confirm that MLZ also shares part of his ancestry with the early Eurasian population that later split into European and Asian groups. Although MLZ and Magdalenians share most of their ancestry (**Fig. 2b**), we do not find an affinity to Tianyuan in any Magdalenian-associated individual, including the oldest individual from El Mirón cave in northern Iberia. However, when we calculate an analogous f_4-statistic for Mesolithic individuals of the form f_4*(Mesolithic HGs, Kostenki14; Tianyuan, Mbuti)*, the southern Portuguese Mesolithic individual from Moita do Sebastião stands out with the highest positive observed f_4-statistic in Western Europe (non-significant; Z=2.6) (**Fig. 5a**, **Supplementary Table 2.5**). Similar results were obtained when measuring shared genetic drift between Mesolithic individuals and

Tianyuan via f_3-outgroup statistics of the form f_3*(Mesolithic HGs, Tianyuan; Mbuti)*, where Moita do Sebastião showed the highest observed f_3-statistic (**Fig. 5b**, **Supplementary Table 2.15**). From **Fig. 5b** we can conclude that the affinity to Tianyuan cannot be explained by the EHG to WHG cline that spans the Mesolithic in Europe as the observed f_3-outgroup value for Moita do Sebastião is even higher than those of EHGs, who carry more ANE ancestry and also ancestry from Asian populations[49], and thus also show elevated positive f_3-statistics (lighter colors in **Fig. 5b**). This finding highlights the genetic legacy of the first early Eurasians in southern Europe, and a genetic connection with the Tianyuan individual, which can be traced in European individuals for more than 30,000 years.

6. Exploration of Basal Eurasian and Near Eastern ancestry

The results of the f_4-statistics shown in **Fig. 4a** indicates that MLZ shared more drift with Villabruna and Natufians than with Kostenki14, whilst showing a slightly higher attraction to Natufians (**Supplementary Table 2.10**). On the basis of this observation we concluded that MLZ carried excess Near Eastern ancestry that was not present in other preceding Gravettian-associated individuals from central Europe or in the Villabruna individual itself. However, an f_4-statistic of the form f_4*(Natufian, Villabruna; MLZ, Mbuti)* indicates that MLZ shows its highest attraction to Villabruna-like ancestry (Z=-8.185). This also suggests that the attraction between MLZ and Natufians could be caused by a Villabruna-like ancestry component that is present in Natufians rather than the Basal Eurasian ancestry from the same group (**Supplementary Table 2.11**). Of note, this f_4-statistic could be negative due to an attraction of Natufians to Mbuti, and driven by the Basal Eurasian ancestry present in Natufians. The f_4-statistic above is also negative when Zlatý kůň is used as an outgroup (**Supplementary Table 2.11**), which would remove the effect of outgroup attraction due to the deep ancestry contribution to Natufians.

Feldman and colleagues[50] showed that Natufians can be modeled as a mixture of WHG ancestry and an ancestral component from a Basal Eurasian lineage. In order to distinguish which of the two components (WHG or Basal Eurasian) present in Natufians is creating this attraction, we explored the potential presence of Basal Eurasian ancestry in MLZ in more detail. The concept of a 'Basal Eurasian population' was coined by[51] and refers to an inferred ancestral population that had split before the diversification of other non-African lineages. The Basal Eurasian ancestry was observed as residual in admixed populations, with modern-day Near Easterners harboring the highest amounts[51,52]. Among Pleistocene HG populations,

CHGs[53], Natufians[52], Anatolian HG[50], and North African individuals from Taforalt (Morocco Iberomaurusian)[54] are the groups with high levels of Basal Eurasian Ancestry.

The detection and quantification of Basal Eurasian ancestry cannot be addressed directly due to the lack of this type of ancestry in unmixed form. Yang and Fu[55] suggested that a positive f_4-statistic of the form *f_4(Han, test; Ust-Ishim, Mbuti)* is an indicator of the test population carrying Basal Eurasian ancestry (**Table S2.12**). Here, Ust'Ishim was considered as an outgroup to all Europeans, as it had been suggested that this individual did not contribute genetically to any later population[43]. On the basis of this rationale, positive results are only expected if the test population shows an attraction to Mbuti, which would be explained by a very close relationship between Africans and (non-African) Basal Eurasians soon after the basal split. By applying this test, we obtained significant positive values for CHGs and Natufians (known to carry Basal Eurasian ancestry) and Oase1. In the case of Oase1, positive values can also result from a higher proportion of Neanderthal ancestry given that the split between Neanderthal and modern humans is basal to the split of African and non-African populations, and thus also closer to Mbuti without the necessity of carrying Basal Eurasian ancestry.

Here, we used f_4-statistics similar to Yang and Fu[55], but also exchanged Han with Kostenki14 as this UP individual contributed to all later Europeans but not to the preceding Ust'Ishim individual: *f4(Kostenki14, test; Ust'Ishim, Mbuti)*. Analogous to Yang and Fu's test, positive results are only expected if the *test* populations carried detectable amounts of Basal Eurasian ancestry (**Supplementary Fig. 6a-6b)**. Our results are consistent with the *f_4-statistics* suggested by Yang and Fu[55] (**Supplementary Fig. 6c, Supplementary Table 2.12**), as both failed to provide convincing evidence for the presence of Basal Eurasian ancestry in MLZ. Based on these results, we speculate that the shared drift between MLZ and Natufians or Near Eastern ancestry is created by a WHG-like ancestry source that is more similar to the WHG component in Natufians[50] than the one represented by the Villabruna individual itself.

However, we have already pointed out that Ust'Ishim might not be an extinct, unadmixed lineage, which means that observing negative f_4-statistics could be expected in both tests (**Supplementary Infortamtion 4**). In fact, this is the case for Goyet Q116-1 and MLZ, both of which showed slightly negative values. Of note, negative results are consistent with an ABBA tree conformation that could balance out/counteract a potential BABA conformation that would otherwise be indicative of

Basal Eurasian ancestry. In either case, we did not find any evidence to support traces of Basal Eurasian Ancestry in MLZ.

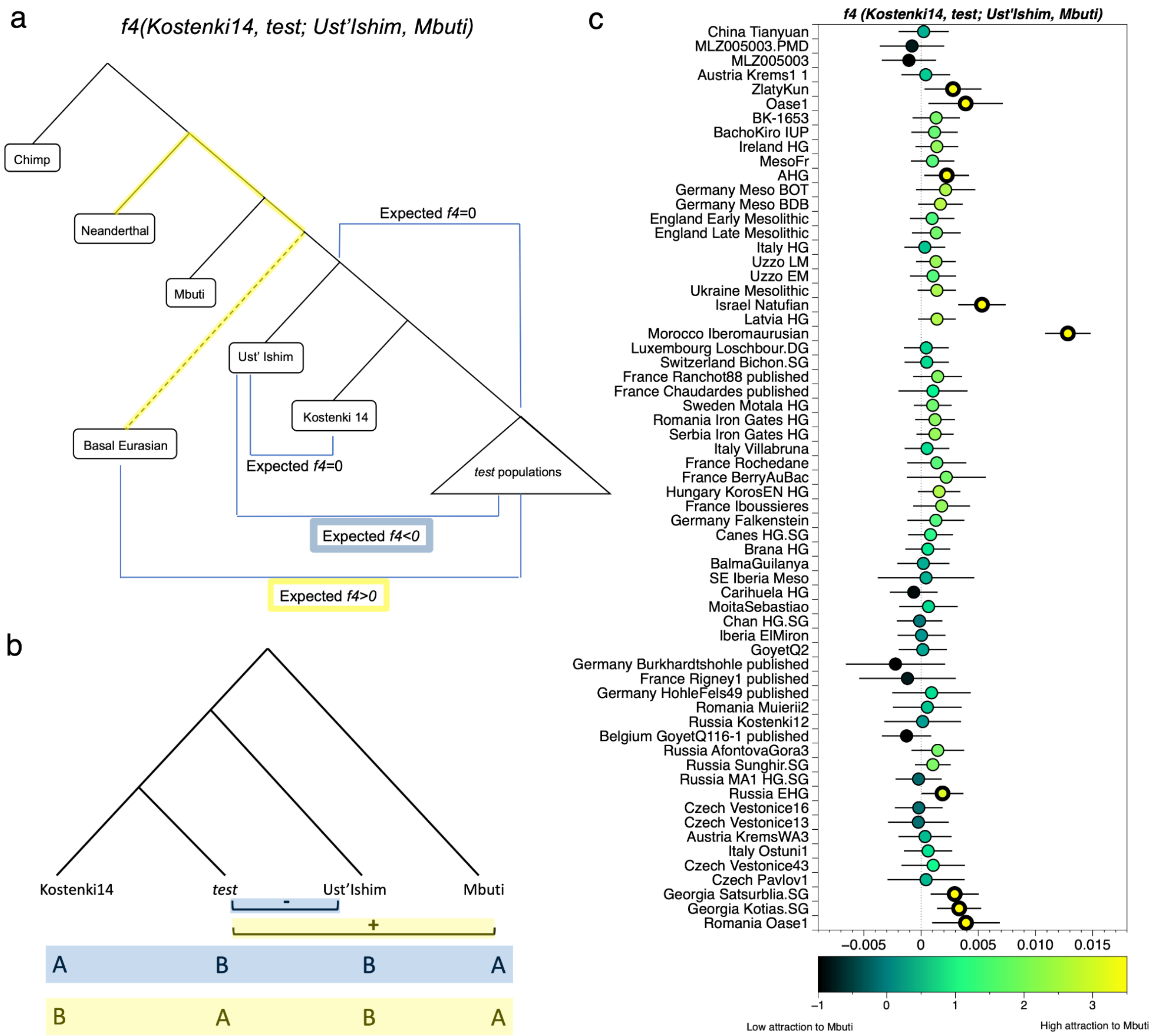

Supplementary Fig. 6 | Testing for Basal Eurasian ancestry. a, Schematic phylogeny that illustrates various shared drift paths of the f_4-statistics used in **Supplementary Fig. 6c.** Here, positive f_4-statistics are only possible when the test population shares ancestry with Mbuti or a Basal Eurasian lineage. **b**, *A visual representation of the interpretation* of the signs of f_4-statistics of the form f_4*(Kostenki14, test; Ust'Ishim, Mbuti)*, **c**, Observed f_4-statistics of the form f_4*(Kostenki14, test; Ust'Ishim, Mbuti)*, where positive values indicate an attraction to Mbuti or to an inferred lineage that is deeper than Ust'Ishim, and which was described as Basal Eurasian ancestry (**Supplementary Table 2.12**). For all f_4-statistics, error bars indicate ± 3 SE and were calculated using a weighted block jackknife [46] across all autosomes on the 1240k panel (nsnps = 1150639) and a block size of 5 Mb.; |Z| > 3 points with thicker outline.

7. Neanderthal ancestry

The South of the Iberian Peninsula was considered to be a region where Late Neanderthals survived longest, coinciding with the Evolved Aurignacian[56–58].

Finlayson et al.[59] dated the survival of Late Neanderthals up to 28 ka cal BP overlapping in time with the Gravettian techno-complex (30-25 ka BP), although such recent radiocarbon dates were subsequently challenged by other scholars[60]. Based on the generally later radiocarbon dates for Late Neanderthals in southern Iberia, and the new MLZ dates, we estimated the proportion of Neanderthal ancestry in the MLZ individual in order to explore if we could find evidence of a higher admixture with Neanderthals.

We calculated the proportion of Neanderthal ancestry[61] using a direct f_4-ratio test (https://github.com/DReichLab, ADMIXTOOLS) of the form Neanderthal ancestry *(alpha) = [f_4(Altai Neanderthal, Chimp; X, Dinka)/f_4(Altai Neanderthal, Chimp; Vindija Neanderthal, Dinka)]*. We did not find a higher amount of Neanderthal ancestry in southern Iberian MLZ than in other Paleolithic individuals from western and central Europe (**Supplementary Fig. 7**, **Supplementary Table 2.13**).

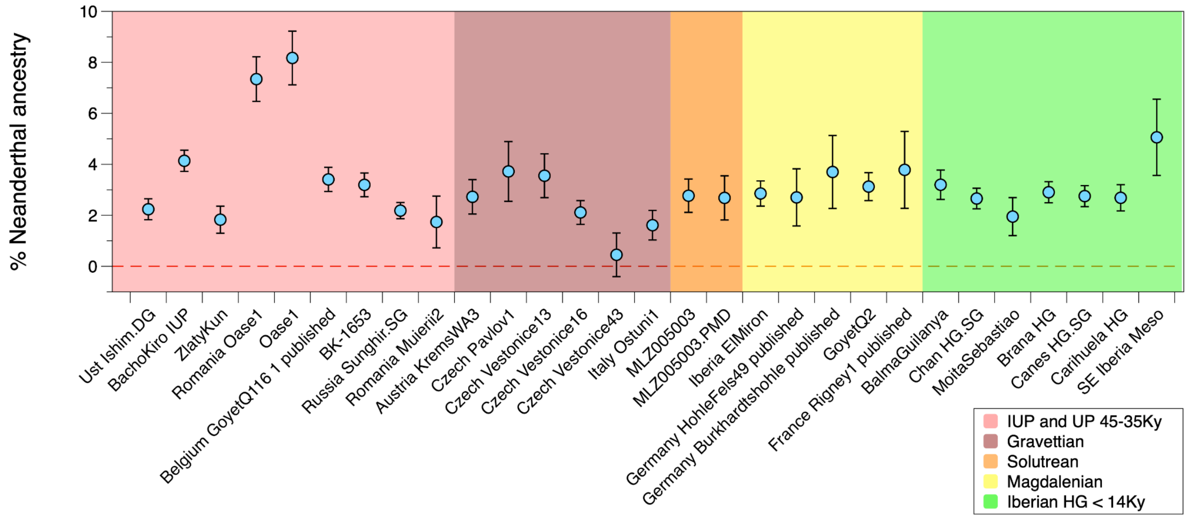

Supplementary Fig. 7 | Percentage of Neanderthal ancestry in Upper Paleolithic individuals calculated following [61] (**Supplementary Table 2.13**). For all f_4-ratio statistics, error bars indicate ± 3 SE and were calculated using a weighted block jackknife[46] across all autosomes on the 1240k panel (nsnps = 1150639) and a block size of 5 Mb.

8. Phylogenetic position of MLZ (qpGraph modeling)

We used *qpGraph* modeling to establish the phylogenetic position of MLZ. We followed a stepwise approach to create a hierarchical framework of ancestral sources represented by prehistoric individuals, including the main West Eurasian HG ancestry lineages, and tested if the ancestry represented by MLZ fits into this framework. In building the *qpGraph* model we also considered the results obtained from accompanying f_3 and f_4-statistics, such as i) the excess of shared drift between MLZ

and Goyet Q116-1 compared to Magdalenians, ii) subtle Early Asian ancestry in MLZ, which was not found in later Magdalenians from Iberia, and iii) the presence of an early WHG ancestry that is different from Villabruna and more similar to the one carried by populations in the Near East.

We started from a skeleton graph of four populations, including Mbuti, Zlatý kůň (~45ka cal BP), Tianyuan (~40ka cal BP), Goyet Q116-1 (~35ka cal BP) and Villabruna (~14ka cal BP). We added Zlatý kůň as an outgroup and then gave the model some flexibility by allowing admixture from the ancestral Tianyuan clade and the Goyet Q116-1 clade, resulting in a worst Z-score of Z = -1.1557. (**Supplementary Fig. 8a**). The decision to add a Villabruna-like lineage next was based on the observation that an admixed form of Villabruna-like ancestry contributed to both the Gravettian-associated individuals and to the Solutrean-associated MLZ individual, which implies that the actual Villabruna-like ancestry must have been much older than the Villabruna individual itself. To bridge the temporal gap, we added intermediate nodes to branch out other older lineages, and also added a theoretical node called Basal_WHG.

We continued with the tree represented by **Supplementary Fig. 8a** by adding MLZ as a result of the admixture between an ancestral node of the Villabruna clade and the sister branch of Goyet Q116-1. This resulted in a worst Z-score of -1.154 (**Supplementary Fig. 8b**)

We continued adding the individual from El Mirón as a representative for the Magdalenian-associated ancestry in the Iberian Peninsula as well as Kostenki14. El Mirón could be fitted as an admixture of Villabruna and MLZ lineages, without any additional contribution with a Z-score = 1.424 (**Supplementary Fig. 8c**). Finally, we added Vestonice16, representing the central European Gravettian as a mixture of Kostenki14 clade and Villabruna-like ancestry (**Supplementary Fig. 9**).

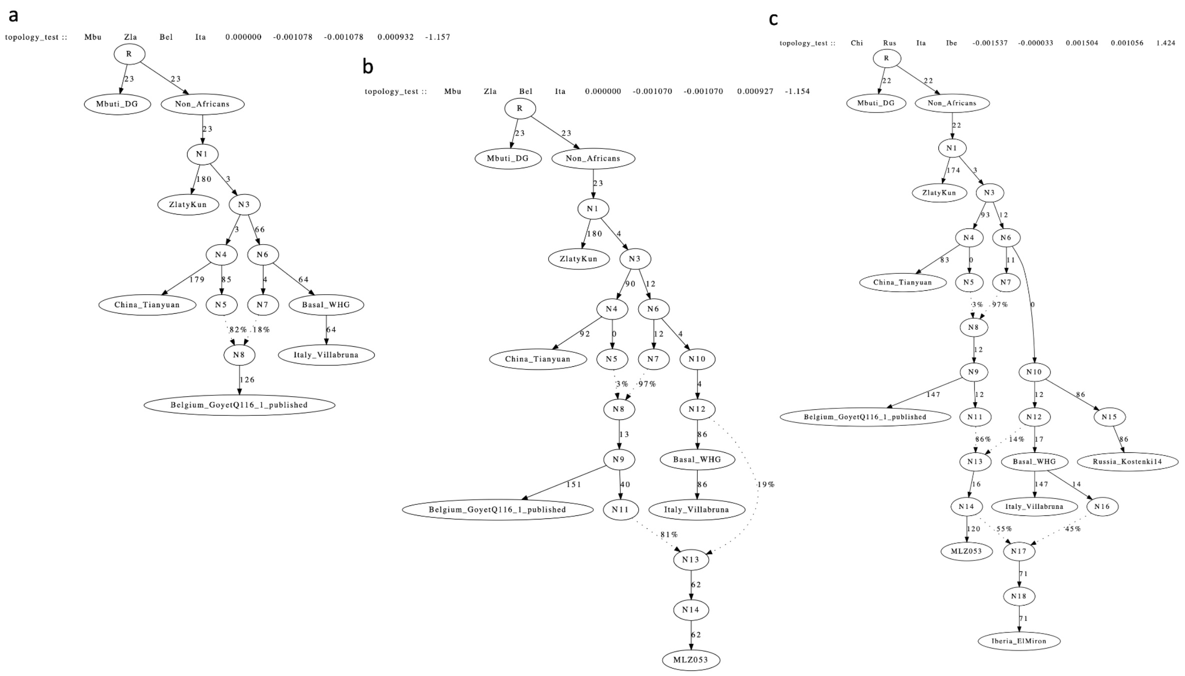

Supplementary Fig. 8 | QpGraph reconstructions including MLZ. a, qpGraph tree fitting Zlatý kůň, Tianyuan, Goyet Q116-1 and Villabruna. **b**, continuation of the same qpGraph tree adding MLZ MLZ005003 as an admixture between ancestry from Goyet Q116-1 clade and Villabruna clade, worst Z-score = -1.154 **c**, continuation of the qpGraph tree fitting El Mirón as an admixture of Villabruna and MLZ lineages, without any additional contribution and Kostenki14, worst Z-score= 1.424.

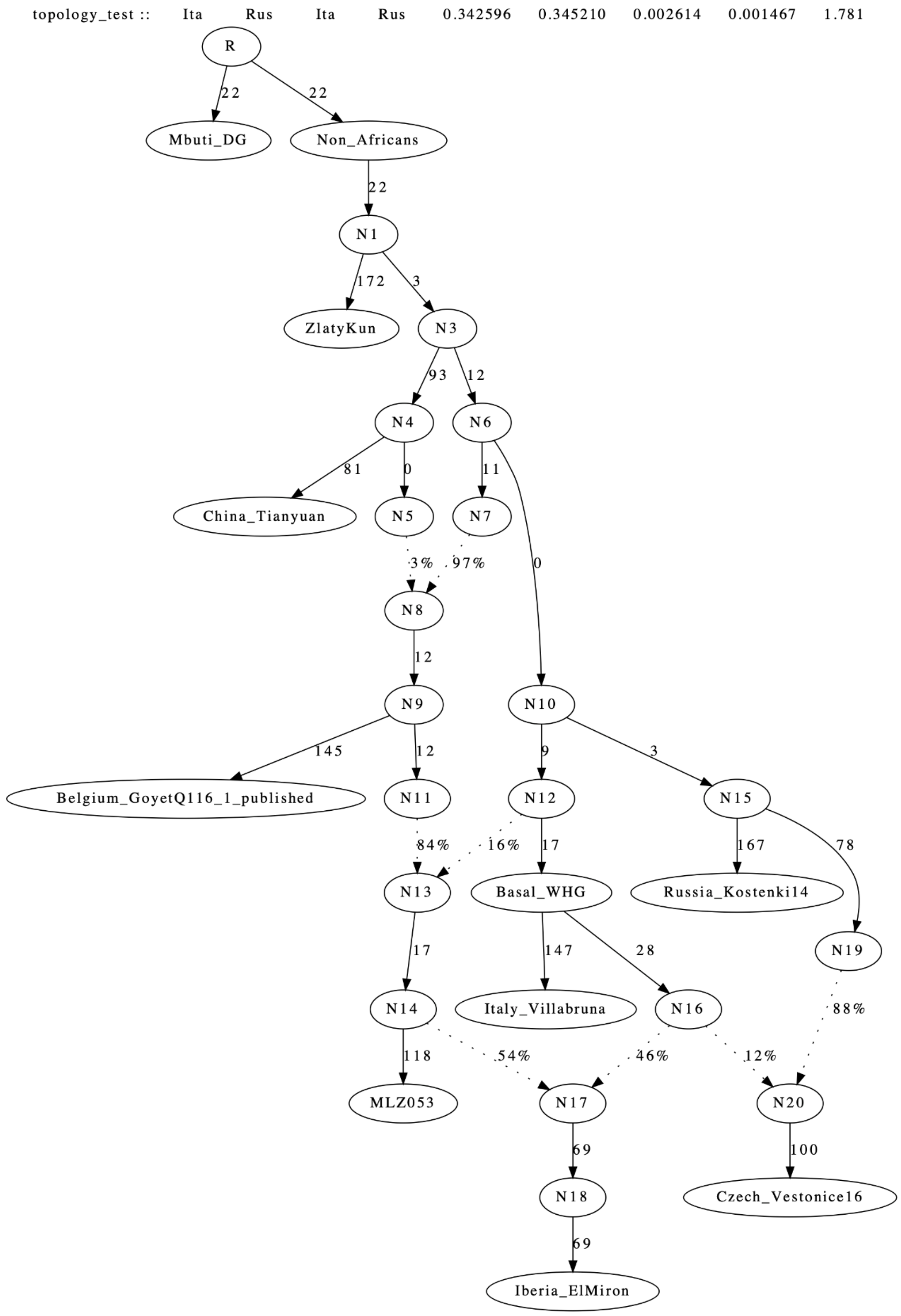

Supplementary Fig. 9 | Final qpGraph reconstruction fitting MLZ and the main ancestral Eurasian sources. Individuals representing the main branches and clusters of genetic ancestry include Zlatý kůň, Tianyuan, Goyet Q116-1, MLZ, El Mirón, Kostenki14, and Vestonice16. Worst Z-score = 1.781.

9. Testing for North African ancestry in MLZ and Moita do Sebastião

Supplementary Fig. 6 already showed that there is no detectable contribution from African or a Basal Eurasian lineage in MLZ or Moita do Sebastião. However, since both individuals come from southern Iberia (although from different time periods) we explored the hypothetical contribution of North African ancestry to MLZ/Moita do Sebastião, or vice versa: MLZ/Moita do Sebastião-related ancestry in North African Morocco Iberomaurusian individuals. To test both scenarios we applied several f_4-statistics. First, we calculated f_4-statistics of the form f_4*(test, Kostenki14, Morocco Iberomaurusian, Mbuti)* to test for shared drift between the test populations and Morocco Iberomaurusian. Observed f_4-statistics were significantly positive for MLZ and Moita do Sebastião and many other WHG and Near Easterns HG groups (**Supplementary Fig. 10a, Supplementary Table 2.16**), which suggests a shared Near Eastern ancestry between Morocco Iberomaurusian and many other HGs, among which MLZ and Moita do Sebastião carry the highest values. We then calculated f_4-statistics of the form f_4*(Morocco Iberomaurusian, Natufian; test, Chimp)* (**Supplementary Fig. 10b**, **Supplementary Table 2.17**) to test if the Near Eastern ancestry found in MLZ, Moita do Sebastião and many other WHGs is linked to Sub-Saharan ancestry, which also contributed to Morocco Iberomaurusian and thus would be a confirmation of an ancestry contribution from Morocco Iberomaurusian. However, significantly negative f_4-statistics for all *test* populations rule out a contribution of Sub-Saharan ancestry, and thus Morocco Iberomaurusian, or at least at much lower levels than in Natufians, which were used as baseline population in the test (**Supplementary Fig. 10b, Table S2.17**).

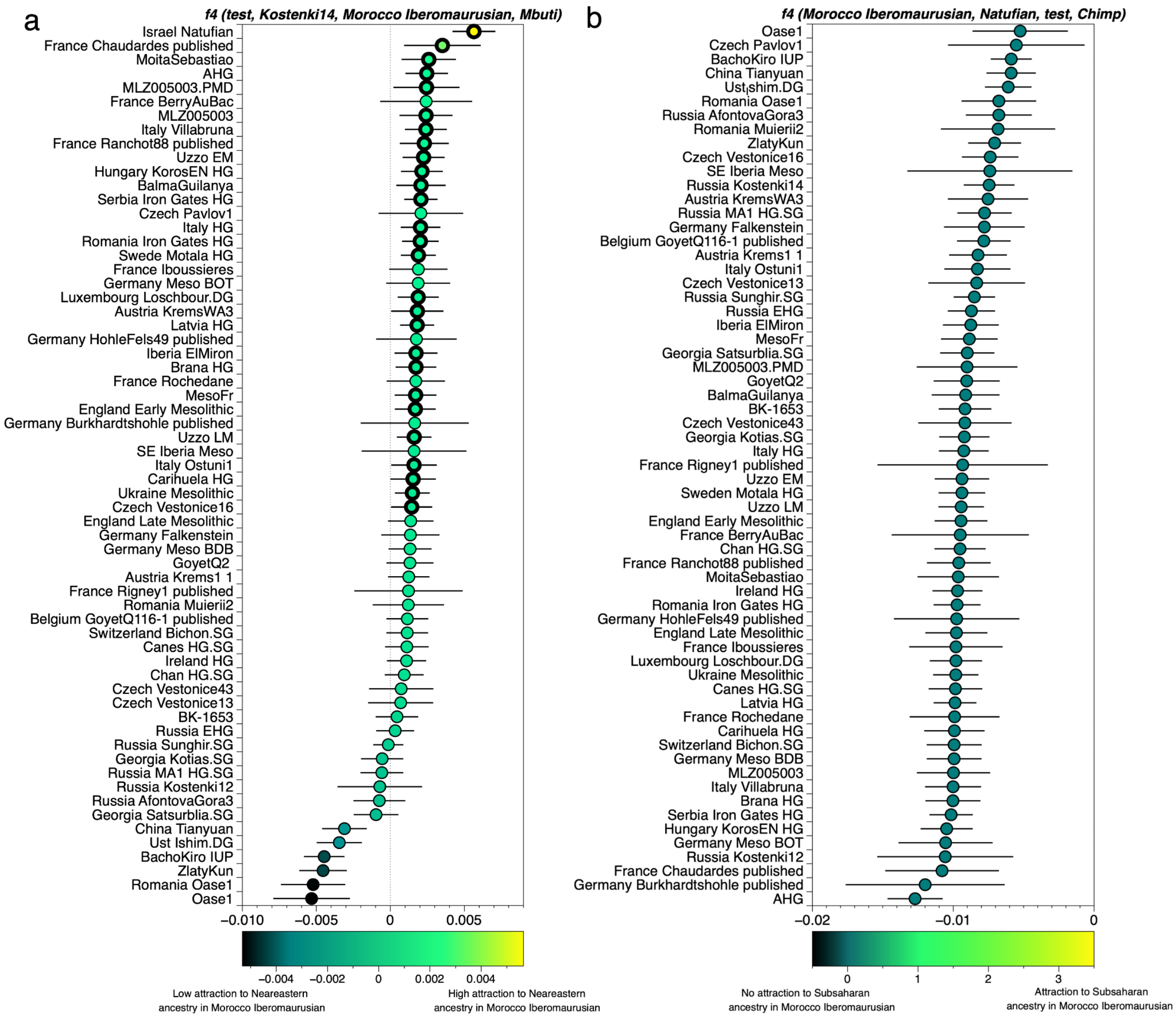

Supplementary Fig. 10 | Exploration of Morocco Iberomaurusian ancestry in other HGs. **a**, f_4-statistics of the form *f4(test, Kostenki14; Morocco Iberomaurusian, Mbuti)* to measure shared drift with Morocco Iberomaurusian when Kostenki14 is used as a baseline (**Supplementary Table 2.16**). **b**, f_4-statistics of the form *f_4(Morocco Iberomaurusian,Natufian; test, Chimp)* to test specifically for shared ancestry with *MoroccoIberomaurusian* by excluding the Near Eastern/Natufian component in Morocco Iberomaurusian and targeting exclusively the Sub-Saharan component as reported in[54] (**Supplementary Table 2.17**). For all f_4-statistics, error bars indicate ± 3 SE and were calculated using a weighted block jackknife [46] across all autosomes on the 1240k panel (nsnps = 1150639) and a block size of 5 Mb.; |Z| > 3 points with thicker outline.

10. Neolithic individuals

In PC space, we noticed a slightly shifted position of Southern_Iberia_EN towards negative values of PC1 and positive values of PC2 (**Supplementary Fig. 11a**). Following this observation, we divided Iberia_EN individuals into two geographic groups, Southern_Iberia_EN (with the exclusion of individual Murcielagos.SG) and Northern_Iberia_EN (**Supplementary Table 2.18**). Our hypothesis to explain the different positions on the PCA plot were as follows:

1. Southern_Iberia_EN carry more HG-like ancestry than Northern_Iberian_EN,
2. Southern_Iberia_EN and Northern_Iberian_EN differ in the type of HG-like ancestry, and/or;
3. Southern_Iberia_EN and Northern_Iberian_EN differ in their distal Neolithic ancestry, where Southern_Iberia_EN are largely derived from the Mediterranean wave of expansion as suggested by other genetic studies, e.g.,[62], and characterized by a subtle proportion of Iran_N-like ancestry[63].

To formally test these hypotheses we used f_4-statistics and *qpWave/qpAdm* following strategies described below, in which we *iterated through various sources* and *rotated these into outgroups*.

10.1 Absence of North African ancestry in Southern Iberia EN

Despite the fact that the position of Southern_Iberia_EN in PCA space does not indicate a North African genetic influence, we nevertheless tested formally whether these groups carried subtle signals of North African ancestry directly with f4-statistics of the form f_4(Southern_EN_indiv, Northern_Iberia_EN; Morocco_EN/LN, Mbuti) (**Supplementary Fig. S11a-11b, Supplementary Table 2.18**). Our results showed that there is no excess shared genetic drift between Southern_Iberia_EN and contemporaneous Moroccan populations compared to Northern_Iberia_EN.

10.2 Exploration of HG ancestry and the potential legacy of different Neolithic waves contributing ancestry to Southern Iberia EN

We used *qpWave/qpAdm* (https://github.com/DReichLab) to quantify the proportion of selected ancestral **sources** in a **target** group. The estimated proportions and the significance of the model differ based on the set of **outgroups** (also known as *right populations*) that are used to differentiate the **sources** used in the model. Here, *qpWave* is used to differentiate sources based on a set of outgroups. We will assume that *qpWave* can distinguish efficiently among the sources when the *qpWave* p-value is < 0.05. Otherwise, *qpAdm* is used to estimate the proportions of each

distinguished source in the target group. However, *qpAdm* behaves like *qpWave* when the **target** has been added as another **source** (both known as *left populations*). In this scenario, *qpWave* outgroups cannot distinguish well between ***sources*** and the ***target*** (included now also as a source) which is in fact an admixture of the other sources. This is the reason why in *qpAdm* we accept models with p-values > 0.05, as it means that the **target** group can be successfully modeled (or better: is not rejected) with the **sources** used in that specific model and based on the set of **outgroups** chosen.

Regarding *qpAdm*, several approaches have been used. The classical approach *iterates through sources.* and retains the models which return a good fit to model the target group (*qpAdm* p-value > 0.05). Using this approach we look for nested models, i.e. most parsimonious, fitting models with minimum number of sources needed. The alternative approach is known as *rotating strategy*[64]. The aim of this approach is to distinguish the true sources needed in the model by moving them to the list of outgroups. If, by doing, so the associated p-value becomes <0.05, the model is rejected (assumptions violated), which in turn indicates that the rotated population is better suited as a direct source. This strategy is helpful when little is known about the populations that are tested.
Here, we address a specific archeological hypothesis that can be translated into differential sources of ancestry, for which we decided to use the *source iteration approach* and the *rotating strategy* only when the two sources tested in analogous models cannot be distinguished. We chose a set of basic outgroups, including African and distal HG ancestral sources which we deemed representative of the main ancestral components in our *target* groups:

South_Africa_2000BP.SG, Ethiopia_4500BP.SG Ust_Ishim.DG, China_Tianyuan, Italy_Villabruna, Belgium_GoyetQ116_1_published, AHG, CHG, Morocco_Iberomaurusian.

All results are reported in **Supplementary Table 2.19** and **Supplementary Fig. 11d**.

A) Iteration approach to test main hypotheses

To model both Iberian_EN North and South, we explored sources from a pre-selected list, which based on our hypothesis (*MLZ/Jordan_PPNB/Iran_N)*

provided a good model fit. We used Anatolia_N and WHG as fixed sources and iterated through MLZ, Jordan_PPNB and Iran_N individually.

Target: *Iberian_EN_North* and *Iberia_EN_South*
Sources: *Anatolia_Neolithic + WHG + (MLZ/Jordan_PPNB/Iran_N)*
Outgroups: *South_Africa_2000BP.SG, Ethiopia_4500BP.SG Ust_Ishim.DG, China_Tianyuan, Italy_Villabruna, Belgium_GoyetQ116_1_published, AHG, CHG, Morocco_Iberomaurusian*.

We found that *Iberian_EN_North* does not require any third source under this model, and Anatolia_Neolithic and WHG fulfill the ancestral sources needed with a p-value of 0.080. On the other hand, *Iberian_EN_South* did not return a good model fit using the same two-way mixture model (p-value: 0.004). However, the model fit for *Iberian_EN_South* improves when MLZ (p-values: 0.262) or Iran_N (p-value: 0.153) were added as a third source. In order to distinguish the best ancestral source we thus applied the *rotation strategy* described below.

B) *Rotating strategy to distinguish the best source (Iran_N or MLZ)*

We first rotated MLZ from the sources to the outgroups to model *Iberian_EN_North* and *_South* as two or three-way admixture with Iran_N as a third source, or on the contrary, rotated Iran_N the outgroups and included MLZ as a third source instead.

Target: *Iberian_EN_North* and *Iberia_EN_South*
Sources: *Anatolia + WHG + (Iran_N)*
Outgroups: *South_Africa_2000BP.SG, Ethiopia_4500BP.SG Ust_Ishim.DG, China_Tianyuan, Italy_Villabruna, Belgium_GoyetQ116_1_published, AHG, CHG, Morocco_Iberomaurusian, MLZ*.

Target: *Iberian_EN_North* and *Iberia_EN_South*
Sources: *Anatolia + WHG + (MLZ)*
Outgroups: *South_Africa_2000BP.SG, Ethiopia_4500BP.SG Ust_Ishim.DG, China_Tianyuan, Italy_Villabruna, Belgium_GoyetQ116_1_published, AHG, CHG, Morocco_Iberomaurusian, Iran_N*.

When MLZ was added as an outgroup, we observed that none of the previous models were supported, including those for *Iberian_EN_North.* This suggests that MLZ ancestry is critical in both target groups (northern and southern). In contrast, when Iran_N was added to the outgroups and MLZ used as a source, we still obtained a good fitting value for the three-way model which includes MLZ as a third source, both in *Iberian_EN_North* and *_South*. We also observe that the estimated proportion of MLZ ancestry is bigger in *Iberia_EN_South* ((12.5% ± 3) of the total ancestry profile) than in *Iberian_EN_North* ((6.5% ± 1.9) of the total ancestry profile). The smaller ancestral proportion of MLZ-like ancestry found in *Iberian_EN_North* also explains why the nested two-way model including only Anatolia_Neolithic and WHG was sufficient to obtain a good initial model fit with *qpAdm* in the iteration approach. Taken together, we found that the total amount of HG-like ancestry (WHG + MLZ) is larger in the South.

C) Iteration through sources to evaluate different Solutrean, Magdalenian or Mesolithic ancestries.

We aimed to distinguish between Solutrean, Magdalenian or Southern Iberian Mesolithic ancestries that contributed to the HG-like ancestry carried by the Iberian EN groups. As the three HG sources are very similar, we iterated through the three potential sources of ancestry as described below:

Target: *Iberian_EN_North* and *Iberia_EN_South*
Sources: *Anatolia_Neolithic + WHG + (MLZ/El Mirón/Moita do Sebastião)*
Outgroups: *South_Africa_2000BP.SG, Ethiopia_4500BP.SG Ust_Ishim.DG, China_Tianyuan, Italy_Villabruna, Belgium_GoyetQ116_1_published, AHG, CHG, Morocco_Iberomaurusian, Iran_N*.

By applying this model, we obtained a good model fit value using any of MLZ, El Mirón or Moita do Sebastião. We interpreted this result as having reached the limit of the resolution of the data due to the similarity of the three sources. Chronologically it is clear that the only population who could have been in direct contact with the EN farmers was the Mesolithic population, but the fact that all three models worked well highlights the higher HG population continuity in Iberia, particularly in the South.

D) ***Rotating strategy to distinguish the best source (MLZ, El Mirón, Moita do Sebastião)***

Finally, in order to gain more power of resolution to distinguish between Soutrean, Magdalenian or Southern Iberian Mesolithic ancestries as potential sources, we rotated each of them to the outgroups while keeping the others as sources.

Target: *Iberian_EN_North* and *Iberia_EN_South*
Sources: *Anatolia_Neolithic* + *WHG* + *(MLZ/El Mirón/Moita do Sebastião)*
Outgroups: *South_Africa_2000BP.SG, Ethiopia_4500BP.SG Ust_Ishim.DG, China_Tianyuan, Italy_Villabruna, Belgium_GoyetQ116_1_published, AHG, CHG, Morocco_Iberomaurusian, Iran_N, (MLZ/EL Mirón/Moita do Sebastião).*

We obtained a good model fit for every source-outgroup combination. The fact that all three models were well supported highlights the higher proportion of HG contribution, and thus a continuation of this ancestry in Iberia, and in particular in the South.

10.3 Exploration of individual ADS007.

The presence of 'steppe-related ancestry' was confirmed for individuals ADS007 by obtaining a more negative f_4-statistic in tests of the form f_4*(Anatolia, Yamnaya_Samara, test, Mbuti)*, in line with other individuals from Bronze Age Iberia (**Table S2.20**). 'Steppe-related ancestry' arrived in Southern Iberia ~2200 cal BCE during the Early Bronze Age[65]. Unfortunately, due to insufficient collagen preservation, we could not obtain a radiocarbon date. Thus, on the basis of the genetic ancestry profile, which indicates the presence of 'steppe-related' ancestry, we tentatively ascribed this individual to the BA or later. While R1b-P312 is the predominant paternal lineage for this time period, we report the Y-chromosome lineage I2a1a1 (**Supplementary Table 1.2**). However, f_4-statistics of the form f_4*(ADS007, SE_Iberia_CA; test, Mbuti),* indicate that Iberian_BA groups shared, in fact, more ancestry with SE_Iberia_CA than with ADS007, which suggests a non-local status for individual ADS007 (**Supplementary Table 2.21**). Finally, we modeled ADS007 as three-way mixture of Anatolia_Neolithic, WHG, and Yamnaya_Samara only when Morocco_Iberomaurusian is removed from the outgroups, which suggests a subtle contribution of Levantine/North African ancestry to ADS007 (**Supplementary Table 2.22**).

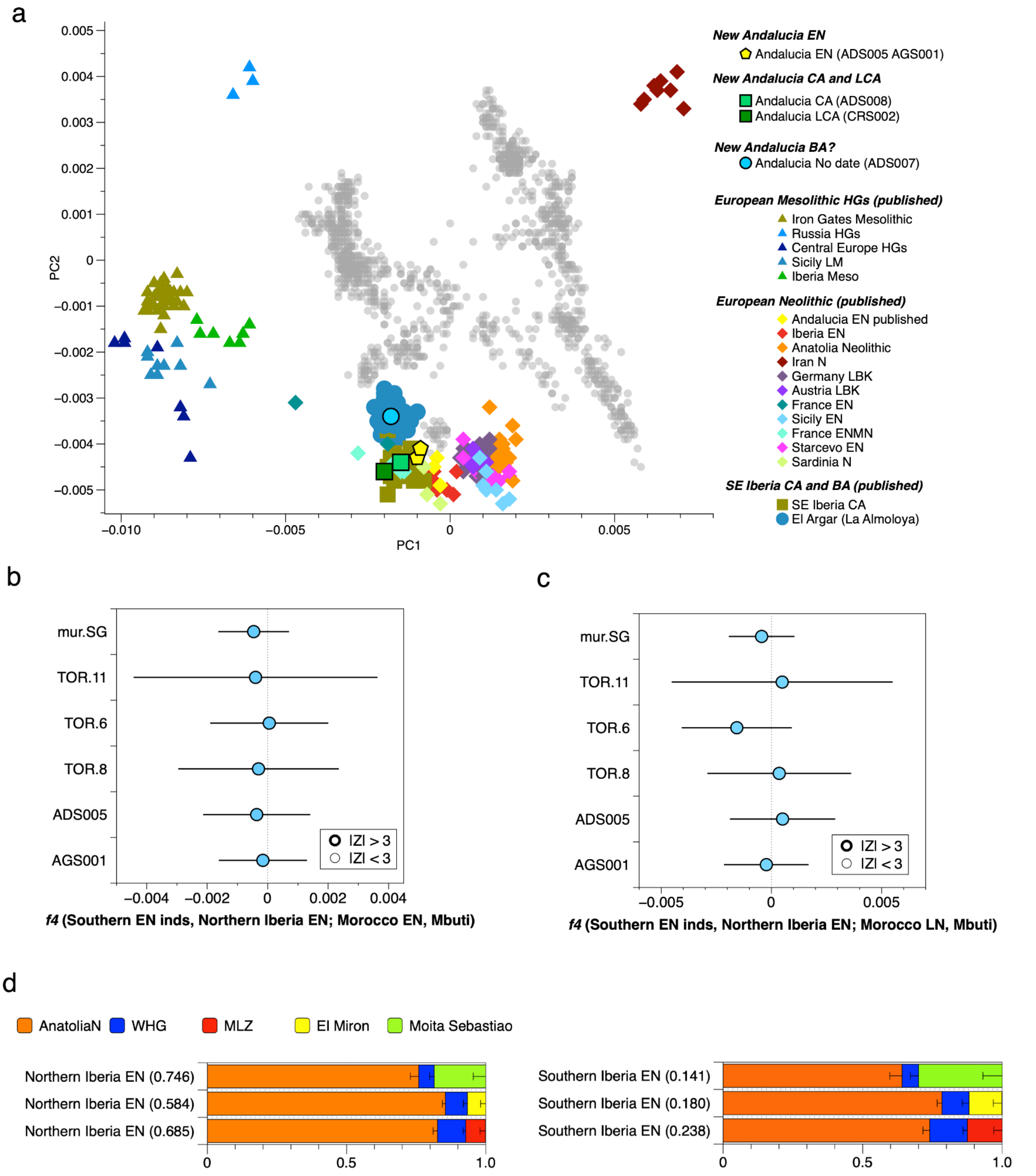

Supplementary Fig. 11 | Analysis of Neolithic, CA and BA individuals: a, West Eurasian PCA calculated with modern populations (grey points), with relevant ancient individuals projected and newly typed individuals with outlines. **b,** f_4-statistics indicating no significant excess of shared genetic ancestry between Southern Iberia EN (Y-axis) and Morocco EN, or **c,** in Morocco LN **(Supplementary Table 2.18)**. Error bars from f_4-statistics indicate ± 3 SE and were calculated using a weighted block jackknife[46] across all autosomes on the 1240k panel (nsnps = 1150639) and a block size of 5 Mb. **d,** admixture model for Southern_EN_Iberia and Northern_EN_Iberian showing the southern group carries higher amounts of Solutrean/Magdalenian-related ancestry (**Supplementary Table 2.19**). Error bars from qpAdm values indicate ± 1 SE.

Supplementary Table 1: Quality analysis and summary of the genomic data
1.1 Paleolithic dataset including all individuals co-analysed in this study
1.2 Archaeological context and main genomic data
1.3 EAGER output of 1240k SNP capture data
1.4 Damage patterns in DNA libraries
1.5 PMD filtering
1.6 PMR results to determine duplicates and infer contamination
1.7 Sex determination estimation
1.8 X-contamination in males
1.9 Mitochondrial contamination
1.10 EAGER output of mitochondrial capture data
1.11 MT-haplogroup assignment

Supplementary Table 2: Detailed genomic data analysis
2.1 f_4-statistics showing the affinity of MLZ to Magdalenian-associated individuals when comparing with Aurignacian-associated Goyet Q116-1.
2.2 f_4-statistics reporting the affinity of Goyet Q116-1 to MLZ when comparing with Madgalenian-associated individuals, and the affinity of Magdalenian-associated individuals to Goyet Q116-1 when comparing with MLZ.
2.3 f_4-statistics showing no significant contribution of ancestry from central European Gravettian-associated individuals to MLZ when compared to Goyet Q116-1.
2.4 f_4-statistics showing no significant contribution of ancestry from central European Gravettian-associated individuals to MLZ when compared to Madgalenian-associated individuals, Goyet Q116-1 and Villabruna.
2.5 f_4-statistics testing for shared drift between MLZ and published HG when Kostenki14 is used as baseline.
2.6 f_4-statistics testing for shared drift between HGs and Tiayuan when Kostenki14 is used as baseline.
2.7 f_4-statistics testing for shared drift between individual test pairs and Tianyuan.
2.8 f_4-statistics to test for shared drift between Ust'Ishim and MLZ.
2.9 f_4-statistics to detect Villabruna ancestry in Magdalenian-associated individuals.
2.10 f_4-statistics to test differential genetic affinity of Pleistocene European HG and Iberian Holocene HG with Natufian and Villabruna-like ancestry.
2.11 f_4-statistics to measure differential genetic attraction among MLZ, Natufians and Villabruna.
2.12 f_4-test to infer Basal Eurasian ancestry.
2.13 Percentage of Neanderthal ancestry in HGs.
2.14 f_4-statistics testing for shared drift between HGs and MLZ when Goyet Q116-1 is used as baseline.
2.15 f_3-output statistics reporting differential affinity between European Mesolithic HG and Tianyuan.
2.16 f_4-statistics to test for shared drift with Morocco Iberomaurusian in all HGs when Kostenki14 is used as a baseline.
2.17 f_4-statistics to test for shared ancestry with Morocco Iberomaurusian in all HGs by excluding the Near Eastern/Natufian component from Morocco Iberomaurusian.
2.18 f_4-statistics to test differential North-African ancestry in southern and northern Iberian neolithic groups.

2.19 *qpAdm* admixture modeling of southern and northern Iberian neolithic groups calculated using ADMIXTOOLS (https://github.com/DReichLab). Target groups, sources, outgroups and p-values are listed in the table.
2.20 f_4-statistics to infer Steppe ancestry in individual ADS007.
2.21 f_4-statistics to infer differential affinity to local or non-local groups by ADS007
2.22 *qpAdm* admixture modeling for the individual ADS007 calculated using ADMIXTOOLS (https://github.com/DReichLab). Sources, outgroups and p-values are listed in the table.

Supplementary references

1. Carrión, F. & Contreras, F. Yacimientos Neolíticos en la zona de Moclín, Granada. *Cuadernos de Prehistoria y Arqueología de la Universidad de Granada* **4**, 21–56 (1979).
2. Cantalejo Duarte. La cueva de Malalmuerzo (Moclín, Granada): nueva estación con Arte Rupestre Paleolítico en el área mediterránea. *Antropología y Paleoecología Humana* **3**, 59–99 (1983).
3. Cabello, L. *et al.* New archaeological data on the upper Paleolithic site of cueva de Malalmuerzo (Moclín, Granada, Spain). *Munibe Antropol.-Arkeol.* (2020) doi:10.21630/maa.2020.71.07.
4. Breuil, H. *Nouvelles cavernes ornées Paléolithiques dans la province de Málaga*. (L´Anthropologie XXXI, 1921).
5. Cantalejo, P. & Espejo, M. M. Patrimonio prehistórico en el sur de la Península Ibérica. in *Pleistocene Foragers on the Iberian Peninsula. Their Culture and Environment. Festschrift in honour of Gerd-Christian Weniger for his sixtieth birthday.* (eds. Pastoors, A. & Auffermann, B.) vol. 7 101–117 (Mettmann: Neanderthal Museum Wissenschaftliche Schriften, 2013).
6. Cantalejo, P. *et al. La Cueva de Ardales: Arte prehistórico y ocupación en el Paleolítico Superior*. (Málaga: CEDMA., 2006).
7. Sanchidrián Torti, J. L. Arte paleolítico de la zona meridional de la Península Ibérica. *Complutum* **5**, 163–195 (1998).
8. Espejo, M. M. & Cantalejo, P. Nuevas aportaciones al Corpus artístico paleolítico del extremo occidental del Mediterráneo. in *Actas del Congreso Internacional del Estrecho*

de Gibraltar (ed. Ripoll, E.) vol. I 131–146 (Madrid: UNED, 1987).

9. Espejo, M. M. & Cantalejo, P. Cueva de Ardales: yacimiento recuperado. *Revista de Arqueología* **84**, 14–24 (1988).
10. Ramos, J. *et al.* Geocronología evolutiva y cambios climáticos en el Pleistoceno Superior y Holoceno. Los testimonios de su ocupación por formaciones sociales de cazadores-recolectores, tribales y clasistas iniciales. *Mainake* 197–261 (1998).
11. Ramos-Muñoz, J. *et al. Cueva de Ardales. Su recuperación y estudio*. (Málaga: Ayuntamiento de Ardales., 1992).
12. Ramos-Muñoz, J., Martín, E., Espejo, M. M., Cantalejo, P. & Recio, A. Las ocupaciones prehistóricas en la Cueva de Ardales. in *Geología y Arqueología Prehistórica de Ardales.* (eds. Cantalejo, P. et al.) 111–123 (. Málaga: Grupo Andaluz del Cuaternario (AEQUA), 1995).
13. Ramos-Muñoz, J. *et al.* La imagen de la mujer en las manifestaciones artísticas de la Cueva de Ardales (Ardales, Málaga). Un enfoque desde la relación dialéctica producción y reproducción social. *Revista Atlántica-Mediterránea de Prehistoria y Arqueología Social* **5**, 87–124 (2002).
14. Cantalejo, P., Espejo, M. M. & Ramos-Muñoz, J. *Cueva de Ardales. Guía Arqueológica*. (Málaga, Ayuntamiento de Ardales., 1997).
15. Cantalejo, P. *et al.* La cueva de Ardales: primeras agregaciones gráficas paleolíticas en la Sala de las Estrellas. *Mainake* **XXV**, 231–248 (2003).
16. Cantalejo, P., Ramos-Muñoz, J., Weniger, G.-C. & Espejo, M. M. *Cueva de Ardales. Cuadernos de Divulgación científica*. (Consejería de Cultura y Patrimonio Histórico. Junta de Andalucía., 2021).
17. Cantalejo, P., Espejo, M. M., Ramos-Muñoz, J. & Weniger, G.-C. *Guía de la Cueva de Ardales 1821-2021. Bicentenario de su descubrimiento*. (ArdalesTur Ediciones., 2021).
18. Ramos-Muñoz, J., Weniger, G.-C., Cantalejo, P. & Espejo, M. M. *Cueva de Ardales-Intervenciones Arqueológicas 2011-2014*. (Málaga: Ediciones Pinsapar., 2014).
19. Ramos-Muñoz, J. *et al.* Excavations in Solutrean levels of Ardales Cave (Málaga,

España. in *Human Adaptations to the Last Glacial Maximum* (ed. I. Schmidt, J. Cascalheira, N. Bicho, G.-C. Weniger) 171–187 (Newcastle: Cambridge Scholar, 2019).

20. Ramos-Muñoz, J. *et al. Ocupaciones de la Cueva de Ardales y Sima de las Palomas de Teba por sociedades neandertales*. vol. 2 (Bajo Guadalquivir y Mundos Atlánticos, 2020).
21. Ramos-Muñoz, J. *et al.* Ocupaciones paleolíticas por sociedades neandertales y modernas. in *Actualidad de la investigación arqueológica en España III (2020-2021). Conferencias impartidas en el Museo Arqueológico Nacional* 375–392 (Ministerio de Cultura, Madrid, 2021).
22. Hoffmeister, D. *et al.* The investigation of the ardales cave, Spain - 3D documentation, topographic analyses, and lighting simulations based on terrestrial laser scanning. *Archaeol. Prospect.* **23**, 75–86 (2016).
23. Hoffmann, D. L. *et al.* U-Th dating of carbonate crusts reveals Neandertal origin of Iberian cave art. *Science* **359**, 912–915 (2018).
24. Pitarch Martí, A. *et al.* The symbolic role of the underground world among Middle Paleolithic Neanderthals. *Proc. Natl. Acad. Sci. U. S. A.* **118**, (2021).
25. Ramos-Muñoz, J. *et al.* The nature and chronology of human occupation at the Galerías Bajas, from Cueva de Ardales, Malaga, Spain. *PLoS One* **17**, e0266788 (2022).
26. Espejo Herrerías, M. *et al.* Cerro de las Aguillas: necrópolis colectiva de cuevas artificiales. *Revista de Arqueología* **161**, 14–23 (1994).
27. Ramos Muñoz, J. *et al.* La necrópolis colectiva del Cerro de Las Aguilillas (Ardales-Campillos, Málaga). Inferencias socioeconómicas. *Revista Atlántica-Mediterránea de Prehistoria y Arqueología Social* **1**, 159–180 (1997).
28. Ramos Muñoz, J. *et al.* La necrópolis colectiva de cuevas artificiales del II° milenio a.n.e. del Cerro de las Aguilillas (Ardales-Campillos). in *Geología y Arqueología Prehistórica de Ardales* (eds. Cantalejo, P. et al.) 125–148 (Ayuntamiento de Ardales y Grupo Andaluz del Cuaternario, AEQUA, Málaga, 1997).
29. Ramos Muñoz, J., Martín Córdoba, E., Espejo Herrerías, M. M., Cantalejo Duarte, P. &

Recio Ruiz, A. El poblamiento humano prehistórico del V° al II° milenio a.n.e. en la encrucijada de los ríos Turón, Guadalteba y Guadalhorce. in *Geología y Arqueología Prehistórica de Ardales* (eds. Cantalejo, P. et al.) 149–166 (Ayuntamiento de Ardales y Grupo Andaluz del Cuaternario, AEQUA, Málaga, 1995).

30. Ramos Muñoz, J., Espejo Herrerías, M. & Cantalejo Duarte, P. La formación económico social clasista inicial (Milenios III y II a.n.e.) en los entornos de Ardales (Málaga). in *Simposios de Prehistoria de la Cueva de Nerja II y III* 309–320 (Fundación Cueva de Nerja, Málaga., 2004).
31. Rodríguez Vinceiro, F. J. *et al.* Estado Actual de la investigación arqueometalúrgica prehistórica en la provincia de Málaga. *Trabajos de Prehistoria* **49**, 217–242 (1992).
32. Ramos Muñoz, J., Espejo Herrerías, M. & Cantalejo Duarte, P. Morenito-I. Un enterramiento de la edad del Bronce, Ardales (Málaga). in *XIX Congreso Nacional de Arqueología* 409–428 (Universidad de Zaragoza, 1989).
33. Arteaga Matute, O. Tribalización, jerarquización y estado en el territorio de El Argar. *SPAL Rev. Prehist. Arqueol. Univ. Sevilla* 179–208 (1992) doi:10.12795/spal.1992.i1.09.
34. Arteaga Matute, O. Las teorías explicativas de los 'cambios culturales' durante la Prehistoria en Andalucía: Nuevas alternativas de investigación. in *Actas del III Congreso de Historia de Andalucía* 247–311 (Córdoba, 2002).
35. Schubart, H., Pingel, V. & Arteaga Matute, O. *Fuente Álamo: las excavaciones arqueológicas 1977-1991 en el poblado de la Edad del Bronce*. (Arqueología Monografías, Junta de Andalucía, Sevilla, 2001).
36. Ginolhac, A., Jónsson, H., Orlando, L., Schubert, M. & Johnson, P. L. F. mapDamage2.0: fast approximate Bayesian estimates of ancient DNA damage parameters. *Bioinformatics* **29**, 1682–1684 (2013).
37. Skoglund, P. *et al.* Separating endogenous ancient DNA from modern day contamination in a Siberian Neandertal. *Proceedings of the National Academy of Sciences* **111**, 2229 LP–2234 (2014).
38. Mittnik, A., Wang, C.-C., Svoboda, J. & Krause, J. A Molecular Approach to the Sexing

of the Triple Burial at the Upper Paleolithic Site of Dolní Věstonice. *PLoS One* **11**, e0163019 (2016).

39. Kennett, D. J. *et al.* Archaeogenomic evidence reveals prehistoric matrilineal dynasty. *Nat. Commun.* **8**, 14115 (2017).
40. Mittnik, A. *et al.* Kinship-based social inequality in Bronze Age Europe. *Science* **366**, 731–734 (2019).
41. Korneliussen, T. S., Albrechtsen, A. & Nielsen, R. ANGSD: Analysis of Next Generation Sequencing Data. *BMC Bioinformatics* **15**, 356 (2014).
42. Fu, Q. *et al.* A Revised Timescale for Human Evolution Based on Ancient Mitochondrial Genomes. *Curr. Biol.* **23**, 553–559 (2013).
43. Fu, Q. *et al.* The genetic history of Ice Age Europe. *Nature* **534**, 200 (2016).
44. Prüfer, K. *et al.* A genome sequence from a modern human skull over 45,000 years old from Zlatý kůň in Czechia. *Nat Ecol Evol* **5**, 820–825 (2021).
45. Hajdinjak, M. *et al.* Initial Upper Palaeolithic humans in Europe had recent Neanderthal ancestry. *Nature* **592**, 253–257 (2021).
46. Patterson, N. *et al.* Ancient Admixture in Human History. *Genetics* **192**, 1065–1093 (2012).
47. Fu, Q. *et al.* DNA analysis of an early modern human from Tianyuan Cave, China. *Proceedings of the National Academy of Sciences* **110**, 2223 LP–2227 (2013).
48. Yang, M. A. *et al.* 40,000-Year-Old Individual from Asia Provides Insight into Early Population Structure in Eurasia. *Curr. Biol.* **27**, 3202–3208.e9 (2017).
49. Lipson, M. & Reich, D. A Working Model of the Deep Relationships of Diverse Modern Human Genetic Lineages Outside of Africa. *Mol. Biol. Evol.* **34**, 889–902 (2017).
50. Feldman, M. *et al.* Late Pleistocene human genome suggests a local origin for the first farmers of central Anatolia. *Nat. Commun.* **10**, 1218 (2019).
51. Lazaridis, I. *et al.* Ancient human genomes suggest three ancestral populations for present-day Europeans. *Nature* **513**, 409 (2014).
52. Lazaridis, I. *et al.* Genomic insights into the origin of farming in the ancient Near East.

Nature **536**, 419 (2016).

53. Jones, E. R. *et al.* Upper Palaeolithic genomes reveal deep roots of modern Eurasians. *Nat. Commun.* **6**, 8912 (2015).
54. van de Loosdrecht, M. *et al.* Pleistocene North African genomes link Near Eastern and sub-Saharan African human populations. *Science* **360**, 548 LP–552 (2018).
55. Yang, M. A. & Fu, Q. Insights into Modern Human Prehistory Using Ancient Genomes. *Trends Genet.* **34**, 184–196 (2018).
56. Vega Toscano, L. G., Hoyos, M., Ruiz Bustos, A. & Laville, H. La séquence de la grotte de La Carihuela (Píñar, Grenade). Chronostratigraphie et paléoécologie du Pléistocène Supérieur au Sud de la péninsule ibérique. in *L'Homme de Neanderthal. L'environnement.* (eds. Otte, M. & Laville, H.) vol. 2 169–180 (Études et Recherches Archéologiques de l'Université de Liège 29, 1988).
57. Zilhao, J. Chronostratigraphy of the Middle-to-Upper Paleolithic Transition in the Iberian Peninsula. *Pyrenae* 7–84 (2006).
58. Zilhao. The Ebro frontier revisited. in *The Mediterranean from 50,000 to 25,000 BP* (eds. Camps, M. & Szmidt, C.) 291–311 (Oxbow Books: Oxford, 2009).
59. Finlayson, C. *et al.* Late survival of Neanderthals at the southernmost extreme of Europe. *Nature* **443**, 850–853 (2006).
60. Zilhão, J. & Pettitt, P. On the new dates for Gorham's Cave and the late survival of Iberian Neanderthals. *Before Farming* **2006**, 1–9 (2006).
61. Petr, M., Pääbo, S., Kelso, J. & Vernot, B. Limits of long-term selection against Neandertal introgression. *Proc. Natl. Acad. Sci. U. S. A.* **116**, 1639–1644 (2019).
62. Rohrlach, A. B. *et al.* Using Y-chromosome capture enrichment to resolve haplogroup H2 shows new evidence for a two-path Neolithic expansion to Western Europe. *Sci. Rep.* **11**, 15005 (2021).
63. Antonio, M. L. *et al.* Ancient Rome: A genetic crossroads of Europe and the Mediterranean. *Science* **366**, 708–714 (2019).
64. Harney, É., Patterson, N., Reich, D. & Wakeley, J. Assessing the performance of

qpAdm: a statistical tool for studying population admixture. *Genetics* **217**, (2021).

65. Villalba-Mouco, V. *et al.* Genomic transformation and social organization during the Copper Age–Bronze Age transition in southern Iberia. *Science Advances* **7**, eabi7038 (2021).
